# Constructing regulable supports via non-stoichiometric engineering to stabilize ruthenium nanoparticles for enhanced pH-universal water splitting

**DOI:** 10.1038/s41467-024-46750-6

**Published:** 2024-03-29

**Authors:** Sheng Zhao, Sung-Fu Hung, Liming Deng, Wen-Jing Zeng, Tian Xiao, Shaoxiong Li, Chun-Han Kuo, Han-Yi Chen, Feng Hu, Shengjie Peng

**Affiliations:** 1 College of Materials Science and Technology, Nanjing University of Aeronautics and Astronautics, Nanjing, 210016 China; 2 Department of Applied Chemistry, National Yang Ming Chiao Tung University, Hsinchu, 300 Taiwan; 3 Department of Materials Science and Engineering, National Tsing Hua University, Hsinchu, 30013 Taiwan

**Keywords:** Electrocatalysis, Electrocatalysis, Catalytic mechanisms

## Abstract

Establishing appropriate metal-support interactions is imperative for acquiring efficient and corrosion-resistant catalysts for water splitting. Herein, the interaction mechanism between Ru nanoparticles and a series of titanium oxides, including TiO, Ti_4_O_7_ and TiO_2,_ designed via facile non-stoichiometric engineering is systematically studied. Ti_4_O_7,_ with the unique band structure, high conductivity and chemical stability, endows with ingenious metal-support interaction through interfacial Ti–O–Ru units, which stabilizes Ru species during OER and triggers hydrogen spillover to accelerate HER kinetics. As expected, Ru/Ti_4_O_7_ displays ultralow overpotentials of 8 mV and 150 mV for HER and OER with a long operation of 500 h at 10 mA cm^−2^ in acidic media, which is expanded in pH-universal environments. Benefitting from the excellent bifunctional performance, the proton exchange membrane and anion exchange membrane electrolyzer assembled with Ru/Ti_4_O_7_ achieves superior performance and robust operation. The work paves the way for efficient energy conversion devices.

## Abstract (web summary)

Establishing optimal metal-support interactions is crucial for creating efficient catalysts for water splitting. Here, stable Ti–O–Ru interface formation and minimal work function difference between Ru nanoparticles and non-stoichiometric Ti4O7 lead to exceptional bifunctional activity of Ru/Ti4O7 for both HER and OER across pH-universal environments, maintaining stable operation for 500 h.

## Introduction

Water splitting has received extensive concern as the most promising pathway to obtain green hydrogen^1,2^. Membrane electrode assembly (MEA) water electrolysis allows for clean and efficient green hydrogen production^3,4^. Anion exchange membrane (AEM) and proton exchange membrane (PEM) electrolyzers are promising devices for water electrolysis, which rely on platinum (Pt)-based catalysts for hydrogen evolution reaction (HER)^5–7^. Furthermore, PEM electrolyzers also require corrosion-resistant iridium (Ir)-based materials due to the harsh acidic conditions for oxygen evolution reaction (OER)^8,9^. The large-scale consumption of precious metals has resulted in high costs for PEM, hindering the commercialization of PEM water electrolysis^10,11^. Therefore, it is necessary to develop low-cost catalysts with less or non-Pt/Ir based catalysts. Ru is an effective alternative to Pt/Ir due to about 1/5 of the cost of Pt^12^. More importantly, Ru NPs exhibit unique HER and OER bifunctionality advantages in water splitting to reduce the catalyst cost^12–14^. However, the stronger affinity of Ru for the *H intermediate in HER significantly affects reaction kinetics^15^. In the OER process, Ru easily forms soluble Ru^n+^ species (*n* > 4) under the oxidation potentials, thus exhibiting an extremely short lifetime^16,17^. Enhancing the activity and stability of Ru NPs in both HER and OER is a challenge. Among many strategies of catalyst design, loaded catalysts have garnered widespread attention due to the simple synthesis process, low usage of precious metals, ample exposure of active sites, and high atomic utilization efficiency^18,19^. More importantly, sufficient contact between the support and active phase can construct suitable metal-support interactions and stable interface units to regulate the coordination configuration and electronic structure of interface sites, which optimizes the catalytic performance and stability of the active centers^20–22^.

In general, ideal electrocatalyst support needs to maintain stability with high electroconductibility to minimize excessive energy consumption and catalyst dissolution under the operating voltages^23–27^. Among the common stabilized supports, such as TiO_2_^20,28^, MnO_2_^29^, WO_3_^15,30^, MoO_3_^31^, etc., TiO_2_ has attracted much attention due to its excellent corrosion resistance and adjustable valence^20^. However, TiO_2_ usually displays a broad energy band gap as a typical n-type semiconductor material, which gives it poor conductivity and results in limited application in electrocatalysis^32^. Introducing appropriate oxygen vacancies into TiO_2_ through defect engineering can create defect energy levels to improve the conductivity^28,33^. Nevertheless, random oxygen vacancy distribution makes it difficult to adjust defect states accurately and causes potential structural imbalances in semiconductors. Therefore, constructing non-stoichiometrically stable oxide supports with periodically distributed defects can introduce lattice defects with stable structures. Non-stoichiometric titanium oxides (Ti_n_O_2n–1_), such as TiO^34^, Ti_3_O_5_^35^, and Ti_4_O_7_^34^, possess higher electrical conductivity for several orders of magnitude than TiO_2_^28,34,36^. In particular, Ti_4_O_7,_ as a typical non-stoichiometric titanium oxide, has a similar conductivity to that of graphene, which is attributed to the abundant density of state distribution at the Fermi level^37^. In addition, Ti_4_O_7_ exhibits a more stable structure compared to lower-valence titanium oxides such as TiO. More importantly, the precise non-stoichiometric design of the support can obtain the suitable crystal and electronic structure to achieve adaptability with the active species, which is beneficial to improve the intrinsic activity and stability of the active sites.

Herein, the interaction mechanism between Ru nanoparticles and a series of titanium oxides, including TiO, Ti_4_O_7_ and TiO_2,_ was systematically explored. The appropriate metal-support interaction between Ru and Ti_4_O_7_ is achieved, which moderately induces electron enrichment of the Ru sites to inhibit the lattice oxygen mechanism (LOM) of OER and facilitate deprotonation through stable Ti–O–Ru units, which balances the activity and stability of OER. Furthermore, the low interface resistance between Ti_4_O_7_ and Ru initiates the hydrogen spillover mechanism for HER to accelerate reaction kinetics. The exceptional bifunctional activity is confirmed in pH-universal environments. Specifically, in acidic conditions, Ru/Ti_4_O_7_ demonstrates ultra-low overpotentials of 8 mV and 150 mV at 10 mA cm^−2^ for HER and OER, respectively, and maintains durable operation for 500 h. PEM devices assembled with Ru/Ti_4_O_7_ show lower cell voltages and longer stability than those of commercial RuO_2_ ‖ Pt/C. This work provides new insight into the development of rationally supported catalysts and paves the way for the design of energy conversion devices.

## Results

### Principles of carrier design

**Fig. 1:**
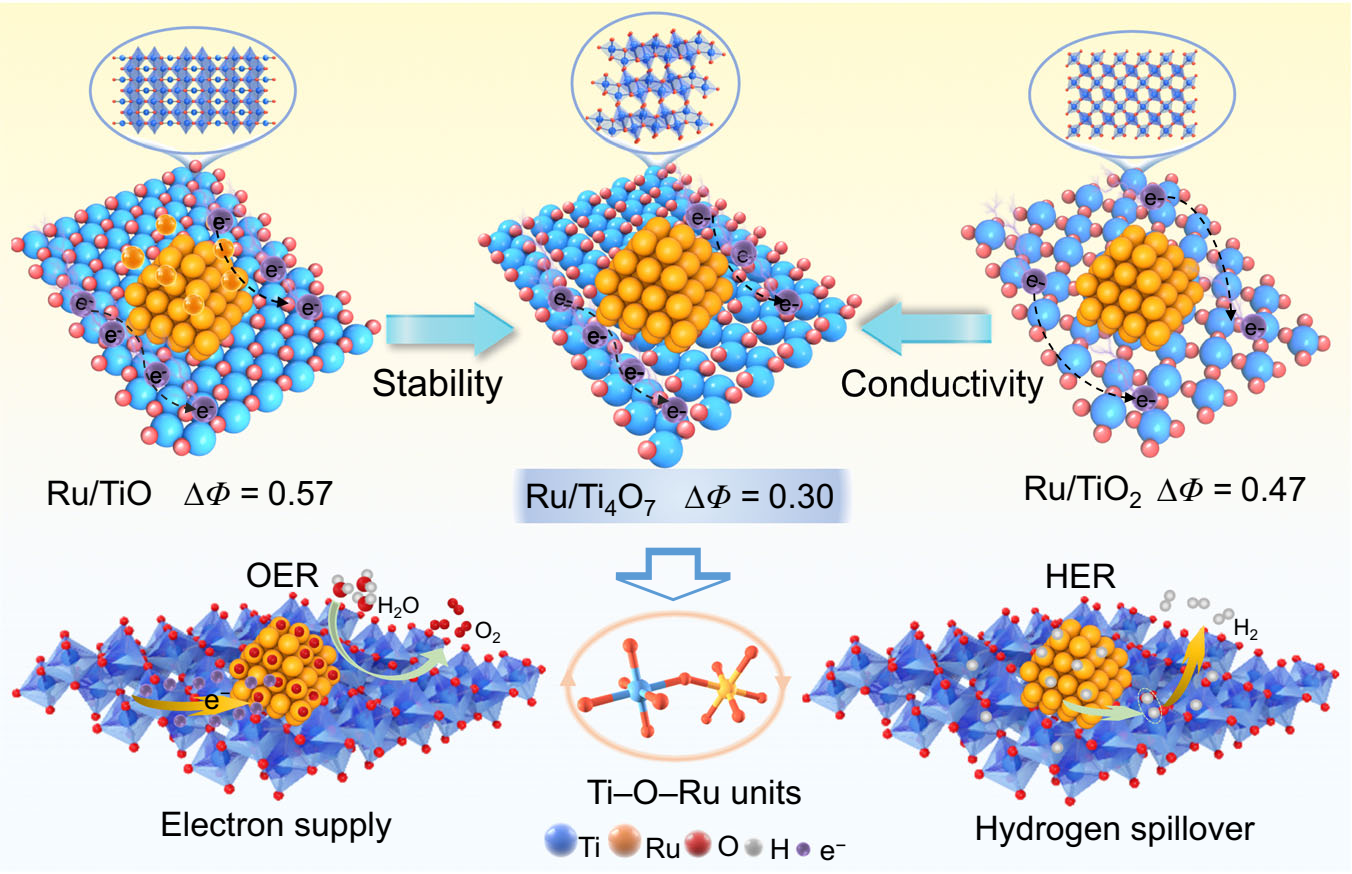
Bifunctional activation based on different supports. Schematic diagram of the interaction mechanism between different titanium oxide supports and Ru NPs to activate OER and HER.

As shown in Fig. 1, non-stoichiometric engineering was employed to finely customize titanium oxide supports (TiO, Ti_4_O_7_, and TiO_2_) with oxygen vacancies of periodic arrangements. Ti_4_O_7,_ with regularly arranged defects, emerges as a potential competitor with superior structural stability over multi-defect TiO and significantly higher conductivity than TiO_2_. More importantly, Ti_4_O_7_ exhibits a suitable metal-support interaction, which lays the foundation for the dual-function activity of Ru/Ti_4_O_7_ in both HER and OER. On the one hand, the higher work function compared to Ru NPs promotes the electron richness of Ru, which can alleviate the dissolution of Ru in OER. On the other hand, compared to TiO and TiO_2_, the minimum work function difference between Ti_4_O_7_ and Ru NPs (∆*Φ* = 0.30 eV) can reduce the interface Schottky barrier (Supplementary Fig. 1), which promotes electron transport in the composite catalyst and trigger hydrogen spillover during HER.

### Morphology and crystal structure

**Fig. 2:**
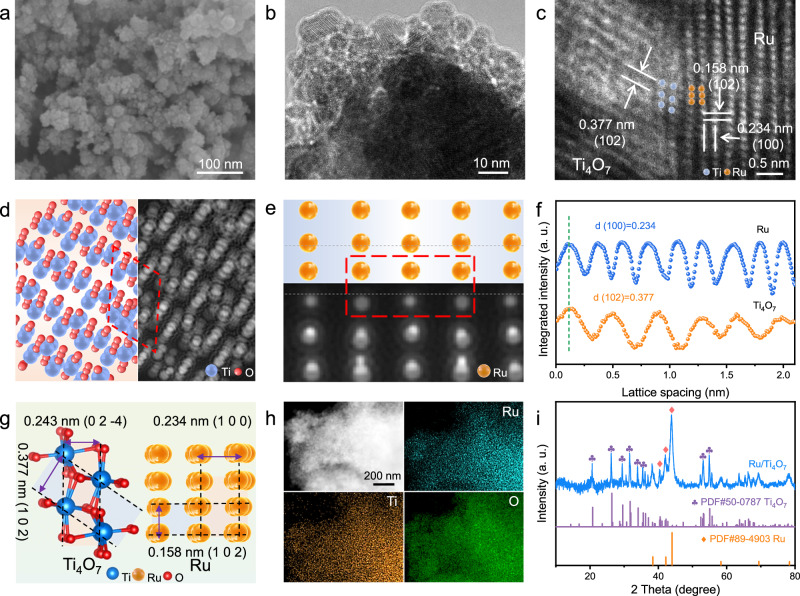
Synthesis and structural characterization. **a** SEM of Ru/Ti_4_O_7_. **b** TEM and **c** AC-HRTEM images of Ru/Ti_4_O_7_. **d**, **e** The schematic atom structure and the corresponding simulated HAADF-STEM images of Ti_4_O_7_ and Ru in Ru/Ti_4_O_7_, respectively. **f** Crystal plane spacing of Ti_4_O_7_ and Ru. **g** Schematic diagram of interface contact between Ti_4_O_7_ and Ru. **h** EDS mapping of Ru, Ti, and O in Ru/Ti_4_O_7_, respectively. **i** XRD pattern of Ru/Ti_4_O_7_.

Ru nanoparticles (NPs) were deposited on titanium oxide supports with different stoichiometric ratios (TiO_2_, Ti_4_O_7_, and TiO) via a wet chemical method (Fig. 2a). Ti_4_O_7_ has potential electrocatalytic advantages in support of Ru NPs due to the chemical stability superior to TiO and electrical conductivity far superior to TiO_2_. Scanning electron microscopy (SEM) and transmission electron microscopy (TEM) images display the uniform coating of the titanium oxide supports by Ru NPs with a size of ~10 nm (Figs. 2b and 1c). The suitable oxygen vacancies in Ti_4_O_7_ promote the Ru^3+^ adsorption on Ti_4_O_7_, which endows the Ru NPs with a more uniform distribution on Ti_4_O_7_ compared to TiO_2_ with low vacancies (Supplementary Figs. 2–6)^38,39^. In aberration-corrected high-resolution (AC-HRTEM) of Ru/Ti_4_O_7_, tight binding and appropriate matching at the nanoscale between the different phases can be observed, accompanied by a smooth transition at the interface (Fig. 2d). The simulated High Angle toroidal dark field image-scanning transmission electron microscope (HAADF-STEM) images obtained through the crystal structure of Ti_4_O_7_ and Ru show remarkable agreement with the experimental results (Figs. 2e and 1f). Based on these atomic images, the lattice spacing of 0.377 nm can be attributed to the (1 0 2) plane of Ti_4_O_7_ (JCPDS 50-0787), while the lattice spacing of 0.234 nm corresponds to the (1 0 0) plane of Ru (JCPDS 89-4903, Figs. 2g, 1h, and Supplementary Fig. 7). In addition, Ru, Ti, and O elements are uniformly distributed in the energy-dispersive spectroscopy (EDS)-mapping of Ru/Ti_4_O_7_ (Fig. 2i), and the characteristic diffraction peaks assigned to Ru and Ti_4_O_7_ coexist in the X-ray diffraction (XRD) pattern (Fig. 2j). The above results indicate that Ti_4_O_7_ can achieve a suitable interfacial lattice contact with Ru NPs, which provides the possibility for adaptive metal-support interaction and rapid interface electron migration^21,40^.

### Electronic and coordination structure analysis

**Fig. 3:**
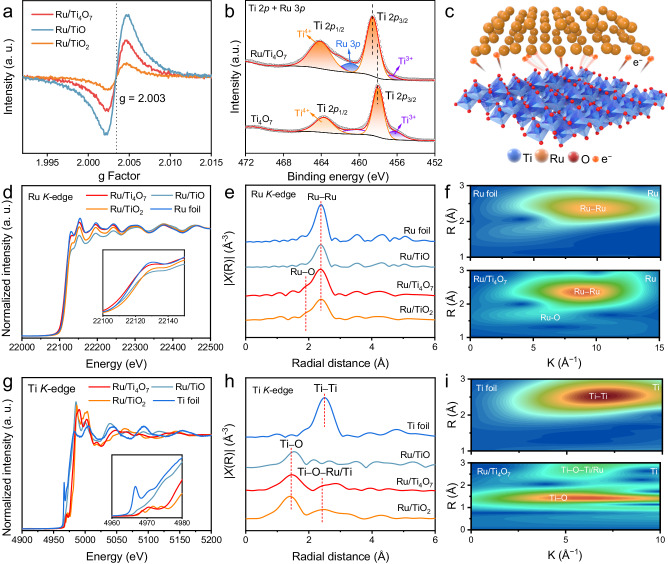
Electron and coordination structure characterization. **a** EPR spectra of Ru/TiO_2_, Ru/Ti_4_O_7_, and Ru/TiO. **b** XPS fine spectra in Ti 2*p* and Ru 3*p* region of Ti_4_O_7_, and Ru/Ti_4_O_7_. **c** Schematic illustration of the electronic interaction between Ti_4_O_7_ and Ru. **d** Normalized Ti *K*-edge XANES of Ti foil, Ru/TiO_2_, Ru/Ti_4_O_7_, and Ru/TiO. **e** The corresponding k^2^-weighted Fourier transforms and (**f**) Wavelet transform of k^2^-weighted EXAFS signals. **g** Normalized Ru *K*-edge XANES of Ru foil, Ru/TiO_2_, Ru/Ti_4_O_7_, and Ru/TiO. **h** The corresponding k^2^-weighted Fourier transforms and **i** Wavelet transform of k^2^-weighted EXAFS signals.

The oxygen vacancy concentration in Ru/TiO_2_, Ru/Ti_4_O_7_, and Ru/TiO shows an increasing trend due to the different stoichiometric ratios of Ti and O (Fig. 3a). The periodically arranged oxygen vacancy not only improves the conductivity of the support but also provides the possibility to form the bridging interfaces between the Ti_4_O_7_ and the Ru NPs. In the X-ray photoelectron spectroscopy (XPS) fine spectra of Ru/TiO_2_, Ru/Ti_4_O_7_, and Ru/TiO, the Ti 2*p* and Ru 3*p* regions display close overlap (Fig. 3b). The binding energy of Ti 2*p* in different Ru-loaded titanium oxide materials shows various degrees of shift to the high binding energy (Fig. 3b, and Supplementary Figs. 8–10), which indicates that titanium oxide can serve as an electron donor to promote electron enrichment of Ru (Fig. 3c). As shown in Supplementary Fig. 8c, Ru NPs exhibit only the characteristic peaks of metallic Ru. In the Ru 3d region of Ru/Ti_4_O_7_, the signals attributed to Ru–O and Ru–Ru can be observed, corresponding to the interface Ti–O–Ru units^41,42^. Similar local electronic changes can be further observed in the fine structure of X-ray absorption (XAFS). In the X-ray absorption near-edge structure (XANES), the Ru *K*-edge also exhibits different white line peak intensity after contacting with various titanium oxide supports (Fig. 3d). Importantly, Ru in Ru/Ti_4_O_7_ has the closest valence state to Ru foil compared with that in Ru/TiO_2_ and Ru/TiO, which is attributed to the low work function difference of Ti_4_O_7_ and Ru obtained by the ultraviolet photoemission spectroscopy (UPS) measurements (Supplementary Fig. 1). Meanwhile, Ti pre-*K*-edge of Ru/Ti_4_O_7_ shows a shift to higher energy compared with that of Ti_4_O_7_, suggesting that the increased valence state of Ti in Ru/Ti_4_O_7_ (Supplementary Fig. 11). The above results indicate that Ru serves as an electron acceptor to form a Schottky contact with the titanium oxide support, and Ti_4_O_7_ possesses the best adaptability of the electronic structure with Ru NPs to reduce the interface Schottky barrier. Extended X-ray absorption fine structure (EXAFS) spectra of Ru corresponding k^2^-weighted EXAFS Fourier transform spectra display a weak Ru–O signal located at 1.92 Å after contacting with TiO_2_ or Ti_4_O_7_ (Fig. 3e). This phenomenon should originate from the interface Ti–O–Ru units between Ru and titanium oxide, which is observed in the wavelet transform more clearly (Fig. 3f). The stable Ti–O–Ru units effectively stabilize the active species and serve as an electron channel to further reduce the interface electron transfer resistance^43^. As shown in Fig. 3g, the shift of Ti pre-*K*-edge reflects the changed oxide valence states of Ti in different stoichiometric titanium oxide supports. Furthermore, As illustrated in the k^2^-weighted EXAFS Fourier transforms of Ti *K*-edge of Ru/TiO_2_ and Ru/Ti_4_O_7_ (Fig. 3h) and the corresponding wavelet transform (Fig. 3i), the structure belonging to Ti–O–Ru/Ti further verifies the formation of the interface Ti–O–Ru units.

### Electrocatalytic activity and stability evaluation

**Fig. 4:**
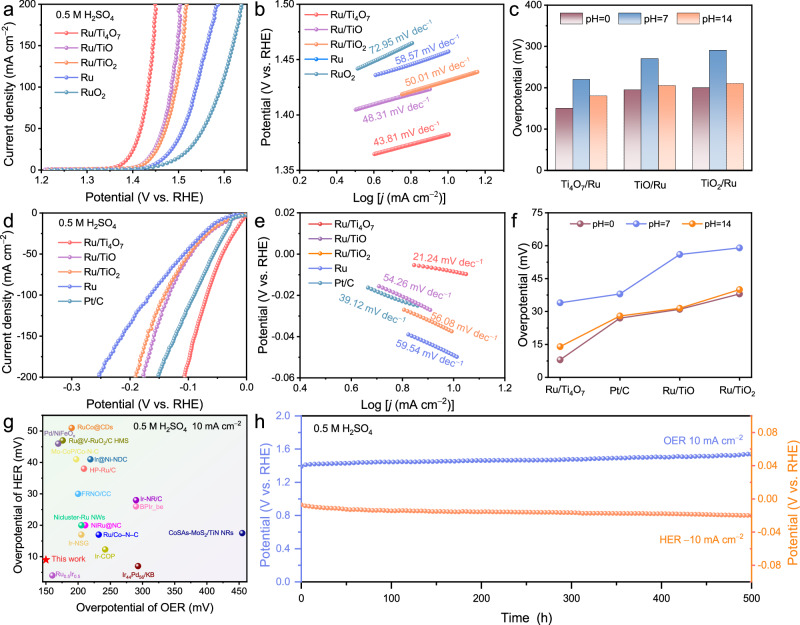
Electrocatalytic activity evaluation. **a** Linear sweep voltammetry (LSV) polarization curves of OER and **b** Tafel plots of Ru, commercial RuO_2_, Ru/TiO_2_, Ru/Ti_4_O_7_, and Ru/TiO (loading amount: 1 mg cm^−2^) in 0.5 M H_2_SO_4_ (pH = 0.3). **c** The corresponding OER overpotential at 10 mA cm^−2^ in different pH environments. **d** LSV polarization curves of HER and (**e**) Tafel plots of Ru, commercial Pt/C, Ru/TiO_2_, Ru/Ti_4_O_7_, and Ru/TiO (loading amount: 1 mg cm^−2^) in 0.5 M H_2_SO_4_ (pH = 0.3). **f** The corresponding HER overpotential at −10 mA cm^−2^ in different pH environments. **g** Comparison of overpotential with that of other bifunctional catalysts for water splitting in 0.5 M H_2_SO_4_ recently reported. **h** Chronopotentiometry curves of Ru/Ti_4_O_7_ at 10 mA cm^−2^, and −10 mA cm^−2^ in 0.5 M H_2_SO_4_.

The electrocatalytic activities of the Ru-loaded titanium oxides were evaluated in a standard three-electrode system to verify the unique advantages of the Ti_4_O_7_ support. In the acidic media, Ru/Ti_4_O_7_ with optimized Ru loading and annealing temperature exhibits the lowest OER overpotential (150 mV at 10 mA cm^−2^, Fig. 4a and Supplementary Fig. 12) and Tafel slope (41.26 mv dec^−1^, Fig. 4b, c). For HER, Ru/Ti_4_O_7_ also displays an ultra-low overpotential of 8 mV at 10 mA cm^−2^ in acidic environments, which is superior to commercial Pt/C and other titanium oxide supported Ru NPs (Fig. 4d). Remarkably, as shown in Fig. 4e, the Tafel slope of Ru/Ti_4_O_7_ with minimum work function difference is only 21.24 mV dec^−1^, which is significantly lower than the value of the conventional Volmer-Heyrovsky/Tafel mechanism (30 mV dec^−1^)^44–46^. The low work function difference between metal and support is prone to trigger the hydrogen spillover mechanism during HER and provide a low interfacial Schottky barrier and fast interfacial electron migration^47^. Electrochemical impedance spectroscopy (EIS) further verifies the lowest electron-transfer resistance of Ru/Ti_4_O_7_ (Supplementary Fig. 13). Notably, the superior difunctional activity of Ru/Ti_4_O_7_ also exhibited scalability in the neutral and basic environments (Fig. 4c, f, and Supplementary Figs. 14–17). In addition, Ru/Ti_4_O_7_ exhibits the highest double-layer capacitance (*C*_dl_) value (68.95 mF cm^−2^) with the same Ru loading, suggesting that more exposed Ru active sites of the Ru/Ti_4_O_7_ compared to Ru/TiO_2_ and Ru/TiO (Supplementary Figs. 18 and 19). The LSV polarization curves normalized by *C*_dl_ and the mass of Ru indicate that the active Ru sites in Ru/Ti_4_O_7_ have the highest specific activities (Supplementary Fig. 20) and mass activity (Supplementary Fig. 21). The outstanding bifunctional performance of Ru/Ti_4_O_7_ exceeds that of most of the excellent bifunctional catalysts in the recent reports (Supplementary Tables 2, 3), especially in acidic environments (Fig. 4g).

The stability of Ru/Ti_4_O_7_ was validated via chronopotentiometry. In severe acidic environments, Ru/Ti_4_O_7_ demonstrates surprising stability of OER and HER for 500 h with a negligible activity decay (Fig. 4h) and slight structural collapse (Supplementary Fig. 22), which exceeds the operating life of Ru/TiO (Supplementary Fig. 23). Ru/Ti_4_O_7_ also requires lower potentials to drive OER compared to Ru/TiO_2_, contributing to the lower energy consumption of the water electrolysis devices over a long period (Supplementary Fig. 23). More importantly, Ru/Ti_4_O_7_ can maintain stable operation for 300 h at a high current density of 200 mA cm^−2^ with only slight activity decay after 3000 cyclic voltammetry (CV) cycles (Supplementary Figs. 24 and 25). The lower current response in the CV corresponding to the oxidation-reduction of Ru in Ru/Ti_4_O_7_ compared to Ru and Ru/TiO implies the inhibited oxidation of Ru by Ti_4_O_7_ (Supplementary Fig. 26). Supplementary Fig. 27 manifests that the Ru and Ti loss of Ru/Ti_4_O_7_ gradually slows down during long-term OER. Ti gradually dissolves in the first 50 h of cycling, which is attributed to the reaction between the unstable surface of the electrode. After 50 h, the dissolution rate of Ti slows down due to the stabilization of the surface of the electrode. Furthermore, the characteristic diffraction peaks belonging to Ru and Ti_4_O_7_ can still be observed in the XRD pattern of Ru/Ti_4_O_7_ after OER without new characteristic peaks compared to the apparent dissolution of Ru/TiO (Supplementary Fig. 28). As shown in the electron paramagnetic resonance (EPR) spectra (Supplementary Fig. 29) and XPS spectra of O 1 *s* before and after stability tests (Supplementary Fig. 30a), a slight decrease in oxygen vacancies in the Ti_4_O_7_ support is observed, indicating the structural stability of the Ti_4_O_7_ support under high current densities. In addition, the almost unchanged XPS signals of Ti and Ru with slight shift towards high binding energy after OER further provide evidence for the good structural maintenance of Ru/Ti_4_O_7_ during the reaction (Supplementary Fig. 30b). The excellent stability of Ru/Ti_4_O_7_ is attributed to inhibited oxidation process, which should be derived from the electron enrichment of Ru through the stabilized Ti–O–Ru units.

### Origin of enhanced difunctional activity of Ru/Ti_4_O_7_

**Fig. 5:**
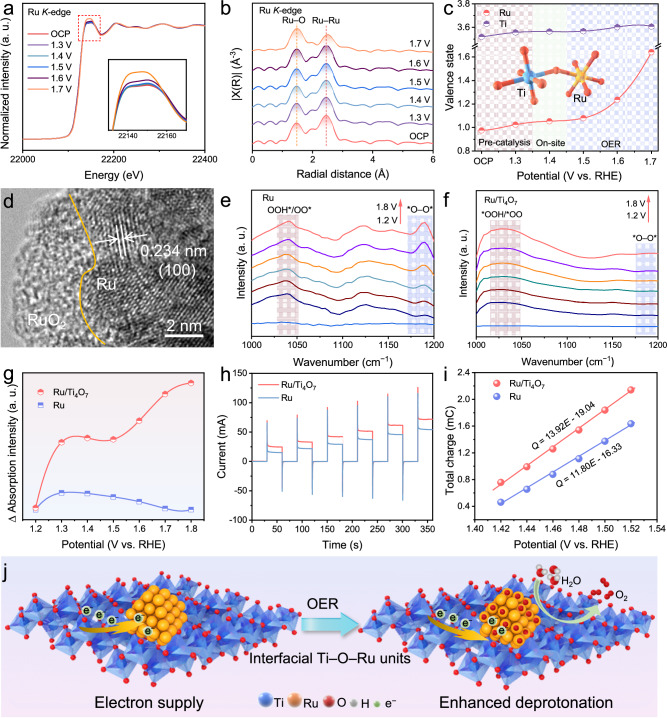
In-situ characterization and reaction mechanism. **a** Normalized in-situ Ru *K*-edge XANES of Ru/Ti_4_O_7_. **b** The corresponding k^2^-weighted Fourier transforms. **c** The valence states of Ru and Ti in Ru/Ti_4_O_7_ obtained via Ru *K*-edge under different potentials. **d** HRTEM of Ru/Ti_4_O_7_ after OER. In-situ ATR-SEIRAS spectra of (**e**) Ru and **f** Ru/Ti_4_O_7_ for OER in 0.5 M H_2_SO_4_ under different potentials vs. RHE. **g** Intensity difference of the infrared signals at 1038 and 1189 cm^–1^. **h** Current responses to pulse voltammetry for Ru and Ru/Ti_4_O_7_. **i** Relationship between charge storage and potential of Ru and Ru/Ti_4_O_7_. **j** Schematic illustration of the optimized OER process induced by the Ti_4_O_7_ support.

A series of in-situ tests were performed in acid environments to trace the origin of the activity and stability of Ru/Ti_4_O_7_. In-situ XAFS explored the structural evolution of Ru/Ti_4_O_7_ during OER. Ru/Ti_4_O_7_ underwent 20 CV cycles to obtain the stable surface before recording the in-situ XAFS spectra. In the most severe acidic environments, the intensity of the white line of the Ru *K*-edge increases with the applied potentials of OER (Fig. 5a), indicating the oxidation of Ru NPs. Furthermore, in the Fourier transform *R*-space (Fig. 5b), the gradually increased signal intensity of the Ru–O path verifies the formation of RuO_*x*_. Notably, the peaks attributed to metallic Ru–Ru can always be observed even under the high potentials of OER, which corresponds to the Ru metallic phase shown in the XRD pattern of Ru/Ti_4_O_7_ after OER. The above results indicate that the RuO_*x*_ species locally formed on the Ru/Ti_4_O_7_ can act as a barrier layer to slow down the further oxidation and dissolution of Ru NPs. The in-situ XAFS spectra show that the Ti *K*-edge of Ti_4_O_7_ almost completely overlaps under the OER potentials without prominent oxidation characteristics, which benefits from the stable structure of Ti_4_O_7_ (Supplementary Fig. 31a). The *R*-space of Ti further verifies that the Ti–O bond in Ti_4_O_7_ does not change significantly during OER (Supplementary Fig. 31b). The stability of Ru/Ti_4_O_7_ can be more verified in the explicit valence states of Ti, and Ru. The valence state corrected by the normalized white line intensity of the corresponding reference of Ti in Ru/Ti_4_O_7_ changes slightly between 3.52 and 3.61 with the application potentials growing (Supplementary Fig. 32). Meanwhile, the average valence state of Ru increases from 0.98 to 1.64. The significant change does not appear until the potential reaches 1.7 V vs. RHE (Fig. 5c). This phenomenon further verifies that the Ru NPs still maintain structural stability under high potentials of OER. The incomplete oxidation of Ru NPs is beneficial to maintaining the outstanding conductivity of the composite material during the electrocatalytic process. In the HRTEM image of Ru/Ti_4_O_7_ after OER, the tight interfacial contact between Ru NPs and Ti_4_O_7_ can still be maintained (Supplementary Fig. 33). In addition, a locally amorphous structure can be observed in the outer layer of Ru NPs in Ru/Ti_4_O_7_ after OER, which should correspond to the RuO_*x*_ species (Fig. 5d).

Moreover, the reaction mechanism of OER was revealed by in-situ attenuated total reflection-surface enhanced infrared absorption spectra (ATR-SEIRAS). Figure 5e shows that, as the bias increases to 1.4 V vs. RHE, in-situ ATR-SEIRAS spectra of Ru exhibit absorption bands around the vibration frequency of 1038 cm^−1^, corresponding to the *OOH/*OO intermediates. Simultaneously, a distinct absorption signal at the vibration frequency of 1189 cm^−1^ gradually strengthens with the application of OER potential, corresponding to *O-O* in the LOM. In-situ ATR-SEIRAS spectra of Ru/Ti4O_7_ appear at similar positions as Ru, attributing to the absorption bands of *OOH/*OO (Fig. 5f). The broadening and shifting of absorption peaks may originate from the transition of the deprotonation process. Furthermore, almost no signal corresponding to *O-O* is observed around the vibration frequency of 1190 cm^−1^. Additionally, the normalized density difference of in-situ ATR-SEIRAS spectra corresponding to *OOH/*OO and *O-O* signals is presented in Fig. 5g to determine the proportion occupied by adsorption evolution mechanism and LOM in the acidic OER process^38^. The higher adsorption density difference implies a higher proportion of adsorption evolution mechanism in the reaction process. In the high potential range, Ru undergoes surface reconstruction to form RuO_*x*_ species, enabling both the adsorption evolution mechanism and LOM to drive OER. Meanwhile, in-situ ATR-SEIRAS spectra of Ru/Ti_4_O_7_ consistently exhibit a higher adsorption density difference than Ru during OER. This phenomenon indicates that Ru/Ti_4_O_7_ tends to drive OER through the adsorption evolution mechanism, which can realize more stable OER compared with the LOM with potential catalyst dissolution. In the pH-dependence tests, compared with Ru NPs, Ru/Ti_4_O_7_ showed less correlation with acidity, which verified that it was more inclined to drive OER with adsorption evolution mechanism (Supplementary Fig. 34). These results demonstrate that Ru/Ti_4_O_7_ tends to drive OER through the surface adsorption evolution mechanism, which can realize more stable OER compared with the LOM with potential catalyst dissolution^38,48^.

Pulse voltammetry test was employed to assess the deprotonation capability of catalysts, which can confirm the source of the enhanced activity of Ru/Ti_4_O_7_ in OER^49,50^. Under different voltage pulses (Supplementary Fig. 35), Ru and Ru/Ti_4_O_7_ exhibit alternating cathodic and anodic current pulses (Fig. 5h). The oxidation charge storage capacity of different catalysts was further measured by integrating the anodic current response to voltage pulses. As shown in Fig. 5i, Ru/Ti_4_O_7_ demonstrates a higher oxidation charge storage capacity compared to Ru NPs, implying that Ru/Ti_4_O_7_ undergoes a faster deprotonation process to form reaction intermediate *O^49^. These findings suggest that the appropriate metal-support interactions between Ti_4_O_7_ and Ru can activate Ru sites by promoting the deprotonation process in OER. Furthermore, in the EIS bode plots, the pre-OER process of Ru/Ti_4_O_7_ results in an uneven distribution of surface charges (Supplementary Fig. 36), which is manifested by a reduction in the frequency peaks within the range of 1.40-1.45 V vs. RHE and a shift towards higher frequencies compared to the broader transition phase peaks of Ru NPs (1.35–1.45 V vs. RHE). This phenomenon suggests that Ru/Ti_4_O_7_ exhibits a faster charge dissipation to accelerate deprotonation during OER to reaction kinetics^51^. In summary, the moderate metal-support interactions between Ru NPs and Ti_4_O_7_ through Ti–O–Ru units can reduce interface Schottky barriers to accelerate electron transfer and achieve electron enrichment at Ru sites to slow down the corrosion of Ru during OER^52–54^. In addition, electronic modulation of active Ru sites facilitates the deprotonation process of OER (Fig. 5j). During the HER process, EIS Nyquist plots at different current densities were recorded and fitted by the equivalent circuit inset of Supplementary Fig. 37a. *R*_2_ reflects the hydrogen adsorption resistance on the material surface in the equivalent circuit^13,44^. Hydrogen adsorption kinetics were quantified by plotting log*R*_2_ versus overpotential and calculating the EIS-derived Tafel slopes. As shown in Supplementary Fig. 37, the reduced slope of Ru/Ti_4_O_7_ of 21.3 mv dec^−1^ compared with that of Ru/TiO_2_ and Ru/TiO indicates the accelerated hydrogen adsorption kinetics, which is related to the hydrogen spillover (Supplementary Fig. 38, and Supplementary Table 4). The above results further demonstrate that Ru/Ti_4_O_7_ achieves fast HER kinetics through a potential hydrogen spillover mechanism.

### Theoretical calculation for intrinsic activity and stability analysis

**Fig. 6:**
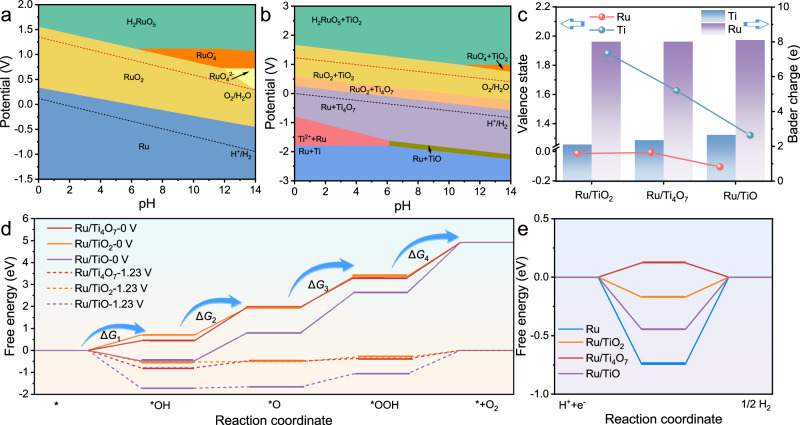
Theoretical calculation and structure-activity relationship. **a** Ru–O, and (**b**) Ti–O–Ru Pourbaix diagrams generated with aqueous ions concentration of 10^−4^ M at 25 °C. **c** Bade charge at the interface of Ru/TiO_2_, Ru/Ti_4_O_7_, and Ru/TiO and corresponding calculated valence states of Ru and Ti. **d** Free energy profiles of different OER intermediates at 0 V and 1.23 V for Ru site in Ru, Ru/TiO_2_, Ru/Ti_4_O_7_, and Ru/TiO. **e** Free energy profiles of HER intermediates for Ru site in Ru, Ru/TiO_2_, Ru/Ti_4_O_7_, and Ru/TiO.

Density functional theory **(**DFT**)** calculations reveal the impact of the support adaptability of Ti_4_O_7_ on the electronic structure modulation and the bifunctional reactivity of the Ru sites. The energy band structure manifests that Ti_4_O_7_ and TiO have a rich density of state distribution at the Fermi level, symbolizing their superior conductivity compared to a noticeable band gap of TiO_2_ (Supplementary Fig. 39). The differential charge density shows a significant yellow area near the Ru atoms at the interface between Ru and Ti_4_O_7_, which verifies the electron enrichment of Ru atoms (Supplementary Figs. 40 and 36c)^55^. In the average potential profile (Supplementary Fig. 41d), the electrostatic potential difference between Ru and Ti_4_O_7_ can be more explicitly observed. Subsequently, Pourbaix diagrams were utilized to verify the stabilization of Ru based on Ti–O–Ru units between Ru and titanium oxide supports. According to the Pourbaix diagram of Ru–O (Fig. 6a), Ru is prone to over-oxidation to form high-valent Ru^4+^ at high potentials for OER, especially in acidic environments. Furthermore, the robust interface Ti–O–Ru units effectively increases the theoretical dissolution potential of Ru in electrochemical oxidation as shown in the Pourbaix system of Ti–O–Ru (Fig. 6b). In Fig. 6c, the Bade and valence state calculations at the interface show that the Ru sites at the Ru/Ti_4_O_7_ interface have the highest valence state (−0.007), which is closest to the initial valence state of Ru and consistent with the XAFS results. This electronic structure adaptability gives Ru/Ti_4_O_7_ a lower interface Schottky barrier compared to Ru/TiO_2_ and Ru/TiO to accelerate interface electron migration and trigger the hydrogen spillover mechanism for HER^47^.

The structural model corresponding to the partially oxidized Ru NPs loaded on Ti_4_O_7_ was constructed to calculate the OER pathways based on the adsorption evolution mechanism (Supplementary Figs. 41–43). The local oxidation combined with the electron redistribution between Ru and different titanium oxide supports endow the Ru sites with varying intrinsic activities through the *d*-band center modulation. The downward shift of the *d*-band center reduces the energy of the antibonding orbitals formed by the adsorption reaction intermediates and the *d*-orbitals of Ru, which implies the weak adsorption of reaction intermediates^48,56^. Therefore, in the surface oxidized Ru/TiO, Ru/TiO_2_ and Ru/Ti_4_O_7_, the *d*-band centers of the Ru sites are −1.80 eV, −1.81 eV and −2.07 eV, respectively (Supplementary Fig. 44 and Supplementary Table 5), which endow the corresponding *OH adsorption free energies with −0.482 eV, 0.435 eV, and 0.703 eV at U = 0 V, respectively (Fig. 6d). Subsequently, the deprotonation process is also optimized. Ru/Ti_4_O_7_ exhibits the smallest deprotonation free energy and the lowest theoretical potential for OER (Δ*G* = 1.540 eV, Supplementary Table 6). The enhanced deprotonation process is consistent with experimental results. The catalysts drive HER in its initial structure at the reduction potentials (Supplementary Fig. 45)^21,57^. The free energy step diagram verifies that the weak HER activity of Ru sites in Ru NPs originated from the strong adsorption of HER intermediates (Fig. 6e). Specifically, the *d*-band center of Ru at the interface exhibits distinct variations with the interface electron migration induced by the contact between Ru and different titanium oxides. The *d*-band center of Ru is −1.72 eV. After contact with TiO, TiO_2_, and Ti_4_O_7_, the corresponding *d*-band centers of Ru shift to −1.77 eV, −1.89 eV, and −1.98 eV, respectively (Supplementary Fig. 46). Owing to the downward shift compared to the original Ru NPs induced by the metal-support interaction, the Ru sites in Ru, Ru/TiO, Ru/TiO_2_, and Ru/Ti_4_O_7_ have *H adsorption free energies of −0.745 eV, −0.446 eV, −0.171 eV, and 0.123 eV, respectively (Fig. 6e, Supplementary Table 7)^58,59^. Therefore, Ru/Ti4O7 has the lowest theoretical reaction potential of 0.123 eV for HER. The theoretical calculations further verified that the appropriate support can promote the intrinsic activity of the Ru sites for both OER and HER.

### Performance of the water electrolysis device

**Fig. 7:**
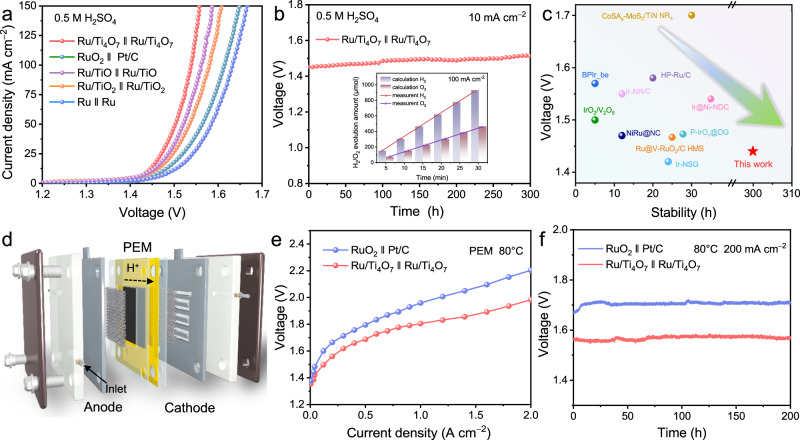
The performance of water splitting. **a** LSV polarization curves of Ru ‖ Ru, RuO_2_ ‖ Pt/C, Ru/TiO_2_ ‖ Ru/TiO_2_, Ru/Ti_4_O_7_ ‖ Ru/Ti_4_O_7_, and Ru/TiO ‖ Ru/TiO (loading amount: 1 mg cm^−2^) in 0.5 M H_2_SO_4_ (pH = 0.3) for the acidic water splitting. **b** Stability tests of Ru/Ti_4_O_7_ ‖ Ru/Ti_4_O_7_ at 10 mA cm^−2^, and the inset display the corresponding Faraday efficiency at 100 mA cm^−2^. **c** Comparison of the cell voltage and stability of Ru/Ti_4_O_7_ ‖ Ru/Ti_4_O_7_ with those of recently reported bifunctional catalysts for acidic water splitting. **d** Conceptual model of the PEM water electrolyzer flow cell using pure water as feedstock. **e** Steady polarization curves of Ru/Ti_4_O_7_ ‖ Ru/Ti_4_O_7_ and RuO_2_ ‖ Pt/C (loading amount: 2 mg cm^−2^) for PEM water electrolyzer in pure water (pH = 6.7) at 80 °C. **f** Stability tests of Ru/Ti_4_O_7_ ‖ Ru/Ti_4_O_7_ for PEM water electrolyzer at 200 mA cm^−2^.

Firstly, the unique advantages of suitable support design were verified through membrane-free water electrolysis tests. As shown in Fig. 7a, the water electrolysis driven by Ru/Ti_4_O_7_ with excellent bifunctional activity only requires a cell voltage of only 1.44 V to reach a current density of 10 mA cm^−2^ with stable operation for 300 h (Fig. 7b) in the acidic environments. Noteworthily, the corresponding Faraday efficiency is also close to 100%. This excellent water splitting performance exceeds that of the state-of-the-art difunctional catalysts recently reported in the acidic media (Fig. 7c). The superiority of the Ti_4_O_7_ support for water splitting in the pH-universal environments has also been verified (Supplementary Figs. 47, 48 and Supplementary Table 8). Benefitting from the advantages of conductivity and stability, Ru/Ti_4_O_7_ was used as both cathode and anode catalysts in the MEA electrolyzers (Fig. 7d). For the PEM water electrolysis (Fig. 7e), Ru/Ti_4_O_7_ ‖ Ru/Ti_4_O_7_ exhibits significantly lower cell voltage than that of RuO_2_ ‖ Pt/C and maintains long-term operation of 200 h at 200 mA cm^−2^ and 300 h at 500 mA cm^−2^ (Fig. 7f and Supplementary Fig. 49), which is superior to most recently reported Ru-based catalysts (Supplementary Table 9). The unique structural advantages of the PEM electrolyzer and the characteristics of high-temperature operation combined with the excellent stability of Ru/Ti_4_O_7_ achieve robust PEM water electrolysis^60^. The EIS Nyquist curves of the MEA further verified the conductivity advantage of Ru/Ti_4_O_7_, which significantly reduces the energy consumption of the electrolyzer under high current densities (Supplementary Fig. 50)^61^. Furthermore, Ru/Ti_4_O_7_ also possesses extensible high activity and stability in AEM (Supplementary Fig. 51). The apparent advantages of bifunctional Ru/Ti_4_O_7_ in membrane electrode assembly show impressive industrialized application prospects.

## Discussion

In this study, titanium oxide designed through rational non-stoichiometric engineering was used to stabilize metal Ru for highly active and stable bifunctional water splitting. The non-stoichiometric design of Ti_4_O_7_ facilitates the achievement of appropriate metal-support interaction with Ru. The electron enrichment of Ru NPs through stable Ti–O–Ru units enhances the corrosion resistance of Ru to obtain partially active RuO_*x*_ species, which inhibits the LOM and accelerates deprotonation for OER. The high electrical conductivity of Ti_4_O_7_ allows Ru/Ti_4_O_7_ to drive OER with low energy consumption. Furthermore, the work function adaptation between Ti_4_O_7_ and Ru makes HER tend to the hydrogen spillover mechanism to achieve fast reaction kinetics. As expected, Ru/Ti_4_O_7_ displays ultralow overpotentials of 8 mV and 150 mV at 10 mA cm^−2^ for HER and OER in acidic environments, respectively, with steady operation for 500 h. The excellent difunctional performance enables Ru/Ti_4_O_7_ to drive water splitting at 10 mA cm^−2^ at a voltage of 1.44 V, which is also validated in pH-universal environments. PEM devices assembled with Ru/Ti_4_O_7_ show lower cell voltages and higher stability than those of commercial RuO_2_ ‖ Pt/C and robust operation at 500 mA cm^−2^ for 300 h. This outstanding performance validates the advancement of delicate support design for water electrolysis devices and opens new avenues for the rational construction of OER catalysts.

## Methods

### Material synthesis

#### Preparation of Ti_4_O_7_

Firstly, 1 g TiO_2_ and 40 mg carbon black were ball-milled for 10 h to form a uniform mixture. Subsequently, the mixture was annealed at 1150 °C for 3.5 h to obtain Ti_4_O_7_ powder.

#### Preparation of Ru/Ti_4_O_7_

Ru-loaded Ti_4_O_7_ was prepared by simple wet reduction. First, 60 mg Ti_4_O_7_ and RuCl_3_ were evenly dispersed in 50 mL of deionized water. Subsequently, 7 mL of 1 M NaBH_4_ solution was slowly added dropwise into the mixture with vigorous stirring. After stirring for 4 h, the product was collected by centrifugation and dried under vacuum at 60 °C for 12 h. The obtained powder was further annealed at 300 °C, 400 °C, and 500 °C for 1 h in the Ar atmosphere. The product was labeled Ru/Ti_4_O_7_ and stored in the Ar-filled environment. The mass of Ti_4_O_7_ was maintained at 60 mg, and the dosage of RuCl_3_ was changed to 20 mg, 40 mg, and 60 mg to achieve different Ru loading.

#### Preparation of Ru/TiO_2_ and Ru/TiO

The preparation and storage methods of Ru/TiO_2_ and Ru/TiO were the same as those of Ru/Ti_4_O_7_. TiO_2_ and TiO replaced Ti_4_O_7_ to obtain Ru/TiO_2_ and Ru/TiO, respectively.

### Material characterizations

XRD tests were carried out utilizing the Bruker D8 advance (Billerica) with a guaranteed scanning rate of 10° min^−1^. SEM images were captured using the Regulus 8100 (Hitachi). AC-TEM (JEM-ARM200F, JEOL) was employed to investigate the fine structure of the materials. The TEM and HRTEM images were acquired using the Tecnai G2 F20 (FEI). XPS utilized Al Kα X-ray as the excitation source (Escalab 250Xi, Thermo Fisher Scientific). All narrow XPS spectra were obtained under the conditions of 20 eV pass energy and 0.05 eV energy step, calibrated by the peak of C 1 *s* located at 284.8 eV. UPS was performed using a VG Scienta R4000 analyzer (monochromatic He I light source of 20.2 eV) with a 10 eV bias. XAFS measurements were conducted at the Taiwan Photon Source 44 A beamline quick-scanning X-ray absorption spectroscopy (Hsinchu). The XAFS spectra were collected at room temperature and analyzed using the Athena program. Inductively coupled plasma-mass spectrometry (ICP-MS) was carried out using the Optima 7300 DV instrument (PerkinElmer).

### Electrochemical measurements

The electrochemical tests were executed using an electrochemical workstation (Autolab PGSTAT302, Metrohm). The potentials were referenced to a reversible hydrogen electrode (RHE). The electrochemical measurements were performed in a three-electrode electrochemical cell with Ar-saturated 0.5 H_2_SO_4_, 1 M KOH aqueous solution, and 1 M phosphate buffered solution (PBS). The active catalysts and binder (Polyvinylidene fluoride, PVDF) were mixed in a weight ratio of 7:1 with N-methyl-2-pyrrolidone (NMP) as the solvent. The catalyst ink was then drop-dried onto the carbon paper and dried for preparation of the working electrode until the catalyst loading was 1 mg cm^−2^. Commercial Pt/C (20 wt. %) and RuO_2_ were prepared as the working electrodes using the same method for comparison. A platinum foil and a carbon rod were used as the counter electrodes for OER and HER, respectively. The Hg/Hg_2_SO_4_, Hg/HgO, and Ag/AgCl electrodes were employed as the reference electrode in acidic, alkaline, and neutral media, respectively. The CV tests at different scan rates were acquired within the 0.6–0.7 V vs. RHE range to determine the *C*_dl_. LSV curves were generated at a scan rate of 5 mV s^−1^ with 85% *iR* compensation. EIS was performed at the potentials of 1.5 V vs. RHE for OER and −0.5 V for HER, covering frequencies from 100 kHz to 0.1 Hz with an amplitude of 10 mV. In the pH-dependence measurement of OER, the electrolyte was prepared by adding the components of Britton-Robinson buffer (0.4 M each of phosphate, borate, and acetate) to a 0.5 M Na_2_SO_4_ solution, and the pH was then adjusted to the desired value by addition of H_2_SO_4_. All glassware was sonicated in ultrapure water directly before electrochemical treatment. Pulse voltammetry was performed while following the current over time. The potential was kept at a low potential (E_l_ = 1.25 V vs. RHE), then switched and kept at a higher potential (E_h_) before returning to E_l_. This cycle was repeated while increasing E_h_ from 1.42 V to 1.50 V in 20 mV/step and keeping E_l_ unchanged. Charges related to the potential step were calculated by integrating the current pulse over time, accounting for the background current signal.

### In-situ electrochemical characterizations

#### In-situ XAFS Measurements

In-situ X-ray absorption spectroscopy, encompassing XANES and EXAFS at both Ru *K*-edge and Ti *K*-edge, was gathered in total-fluorescence-yield mode utilizing a silicon drift detector at the National Synchrotron Radiation Research Center (NSRRC) in Japan and Taiwan. The measurement, under the same conditions as the electrochemical characterization case in a typical three-electrode setup, took place in a specially designed Teflon container with a window sealed by Kempton tape. The scan ranges were kept in an energy range of 22,000–22,400 eV and 4900–5200 eV for Ru *K*-edge (BL-12B2 at SPring-8, NSRRC) and Ti *K*-edge (17C at Taiwan Light Source, NSRRC), respectively. The spectra were obtained by subtracting the baseline of the pre-edge and normalizing that of the post-edge. Fourier transform on k^2^-weighted EXAFS oscillations was employed for EXAFS analysis. All EXAFS spectra are presented without phase correction.

#### In-situ ATR-SEIRAS Measurements

ATR-SEIRAS measurements were performed by a Nicolet iS50 Fourier transform infrared spectrometer (FT-IR) spectrometer with a liquid nitrogen-cooled MCT detector and a fixed angle IR optical path. The spectral resolution of the measurements was 8 cm^−1^, and 32 interferograms were added for each spectrum.

### Membrane Electrode Assembly (MEA) measurements

#### PEM electrolyzer measurements

The membrane electrode assembly was prepared using Nafion 117 by the catalyst-coated membrane method with a geometric area of 1 cm × 1 cm. The bifunctional active catalysts were dispersed in isopropanol, deionized water, and a Nafion ethanol solution (5 wt%) to form an ink. Then, the ink was sprayed on both sides of PEM by the polytetrafluoroethylene (PTFE) transfer with an effective area of 1 × 1 cm^2^ until the catalyst loading was 2 mg cm^−2^. To avoid corrosion at oxidation potential, Ti felt was used as a gas diffusion layer (GDL) for the anode. Finally, the membrane with electrocatalysts coated, the anode GDL, and the cathode GDL (carbon paper) were hot pressed together to establish the MEA under 130 °C with a pressure of 10 MPa for 5 min. The commercial Pt/C (40 wt%) and RuO_2_ were used in the same way as the cathode and anode respectively for comparison. Pure water was sent to the cathode and anode by a peristaltic pump at a speed of 40 rpm. Before testing, the prepared MEA was activated in potentiostatic mode at a cell voltage of 2 V. Subsequently, the electrolyzer was operated at 80 °C, and the hydrogen and oxygen in the electrolyte were removed with Ar to avoid the possible influence of the bubbles formed on the long-term stability of the electrodes. Steady-state polarization curves were obtained by a direct-current power (ITECH, IT-M3223). The cell voltages at different current densities were recorded.

#### AEM electrolyzer measurements

PiperION-A60 was utilized to construct the AEM electrolyzer. The catalyst ink of AEM was the same as that of PEM, except the Nafion ethanol solution was replaced by PiperION A ionomer ethanol (5 wt%). The ink was supported on carbon paper, and Ti felt with the effective area of 1 × 1 cm^2^ until the catalyst loading was 2 mg cm^−2^. To improve the interfacial contact between the catalyst layer and AEM, a small amount of PiperION A ionomer ethanol solution was sprayed on the surface of the anode and cathode catalyst layers and then dried at room temperature for 48 h. Finally, the AEM and the GDLs with catalyst loading were hot pressed together to establish the MEA under 100 °C with a pressure of 10 MPa for 5 min. The commercial Pt/C (40 wt%) and RuO_2_ were also used in the same way as the cathode and anode respectively for comparison. One M KOH aqueous solution was sent to the cathode and anode by a peristaltic pump at a speed of 40 rpm. The electrolyzer was operated at 80 °C.

### Computational methods

All DFT calculations were conducted through the Vienna Ab initio Simulation Package (VASP). The computations utilized the projector augmented wave (PAW)^62^ pseudopotential with the PBE^63^ generalized gradient approximation (GGA) exchange-correlation function. The plane wave basis set had a cutoff energy of 500 eV. K‐sampling in the calculation of adsorption energy used a Monkhorst-Pack mesh of 1 × 1 × 1, while in the calculation of DOS, a 5 × 5 × 1 mesh was employed. Spin polarization was applied to all structures, and complete relaxation of all atoms was ensured with an energy convergence tolerance of 10^−5^ eV per atom. The final force on each atom was maintained below 0.05 eV Å^−1^. Porbaix diagrams were calculated using atomic simulation environment (ASE)^64^ with input formation energy by DFT calculations of bulk and surface models.

In the construction of heterojunctions, the minimum lattice mismatch is used as a criterion to construct the interface contact model between Ru and different titanium oxides. The (0 0 1) facet of TiO contacts the (0 1 0) facet of Ru. The (1 − 1 0) facet of TiO_2_ contacts the (2 1 0) facet of Ru. The (1 1 − 1) facet of Ti_4_O_7_ contacts the (0 0 1) facet of Ru.

The adsorption energy of reaction intermediates can be computed using the following Eq. (1):

1 $$\Delta {G}_{{{{{{\rm{ads}}}}}}}={E}_{{{{{{\rm{ads}}}}}}}-E\ \ast+\Delta {E}_{{{{{{\rm{ZPE}}}}}}}-T\Delta S$$

Where ads = (*H, *OH, *O, *OOH), and (*E*_ads_ − E_*_) is the binding energy, Δ*E*_ZPE_ is the zero-point energy change, Δ*S* is the entropy change. In this work, the values of Δ*E*_ZPE_ and Δ*S* were obtained by vibration frequency calculation.

The Gibbs free energy of the five reaction steps can be calculated by the following Eqs. (2–7):

2 $$ \ast+{{{{{{\rm{H}}}}}}}_{2}{{{{{\rm{O}}}}}}=\ast {{{{{\rm{OH}}}}}}+{{{{{{\rm{H}}}}}}}^{+}+{{{{{{\rm{e}}}}}}}^{-}$$

3 $$*{{{{{\rm{OH}}}}}}=\ast {{{{{\rm{O}}}}}}+{{{{{{\rm{H}}}}}}}^{+}+{{{{{{\rm{e}}}}}}}^{-}$$

4 $$*{{{{{\rm{O}}}}}}+{{{{{{\rm{H}}}}}}}_{2}{{{{{\rm{O}}}}}}=\ast {{{{{\rm{OOH}}}}}}+{{{{{{\rm{H}}}}}}}^{+}+{{{{{{\rm{e}}}}}}}^{-}$$

5 $$*{{{{{\rm{OOH}}}}}}=\ast+{{{{{{\rm{O}}}}}}}_{2}+{{{{{{\rm{H}}}}}}}^{+}+{{{{{{\rm{e}}}}}}}^{-}$$

6 $$*{+{{{{{\rm{H}}}}}}}^{+}+{{{{{{\rm{e}}}}}}}^{-}=\ast {{{{{\rm{H}}}}}}$$

7 $$*{{{{{\rm{H}}}}}}=\ast+1/2{{{{{{\rm{H}}}}}}}_{2}$$

In this work, Δ*G*_1-4_ were calculated at U = 0.

### Supplementary information

Supplementary Information

Peer Review File

### Source data

Source Data File

## Data Availability

The data generated in this study are provided in the Source Data file.

## References

[1] Chong L (2023). La- and Mn-doped cobalt spinel oxygen evolution catalyst for proton exchange membrane electrolysis. Science.

[2] Zhang Y (2023). Hydrogen-bond regulation of the microenvironment of Ni(II)-porphyrin bifunctional electrocatalysts for efficient overall water splitting. Adv. Mater..

[3] Hao S (2021). Torsion strained iridium oxide for efficient acidic water oxidation in proton exchange membrane electrolyzers. Nat. Nanotechnol..

[4] Zheng X (2023). Tailoring a local acid-like microenvironment for efficient neutral hydrogen evolution. Nat. Commun..

[5] Chen Z (2023). Stabilizing Pt single atoms through Pt−Se electron bridges on vacancy-enriched nickel selenide for efficient electrocatalytic hydrogen evolution. Angew. Chem. Int. Ed..

[6] Zhang X-L (2023). Efficient acidic hydrogen evolution in proton exchange membrane electrolyzers over a sulfur-doped marcasite-type electrocatalyst. Sci. Adv..

[7] Hao Y (2023). Methanol upgrading coupled with hydrogen product at large current density promoted by strong interfacial interactions. Energy Environ. Sci..

[8] Shi Z (2022). Enhanced acidic water oxidation by dynamic migration of oxygen species at the Ir/Nb_2_O_5−x_ catalyst/support interfaces. Angew. Chem. Int. Ed..

[9] Ruiz Esquius J (2023). Lithium-directed transformation of amorphous iridium (oxy)hydroxides to produce active water oxidation catalysts. J. Am. Chem. Soc..

[10] Chen M (2023). Recent progress in transition-metal-oxide-based electrocatalysts for the oxygen evolution reaction in natural seawater splitting: a critical review. eScience.

[11] Yang X (2023). IrPd nanoalloy-structured bifunctional electrocatalyst for efficient and pH-universal water splitting. Small.

[12] Li Y (2023). Arming Ru with oxygen-vacancy-enriched RuO_2_ sub-nanometer skin activates superior bifunctionality for pH-universal overall water splitting. Adv. Mater..

[13] Li J (2022). Boosting electrocatalytic activity of Ru for acidic hydrogen evolution through hydrogen spillover strategy. ACS Energy Lett..

[14] Ling C, Shi L, Ouyang Y, Zeng XC, Wang J (2017). Nanosheet supported single-metal atom bifunctional catalyst for overall water splitting. Nano Lett..

[15] Chen J (2022). Reversible hydrogen spillover in Ru-WO_3-x_ enhances hydrogen evolution activity in neutral pH water splitting. Nat. Commun..

[16] Wang N (2023). Doping shortens the metal/metal distance and promotes OH coverage in non-noble acidic oxygen evolution reaction catalysts. J. Am. Chem. Soc..

[17] Zheng X (2022). Ru–Co pair sites catalyst boosts the energetics for the oxygen evolution reaction. Angew. Chem. Int. Ed..

[18] Wei J (2024). Site-specific metal-support interaction to switch the activity of Ir single atoms for oxygen evolution reaction. Nat. Commun..

[19] Monai M (2023). Restructuring of titanium oxide overlayers over nickel nanoparticles during catalysis. Science.

[20] Wu Z (2023). Microwave synthesis of Pt clusters on black TiO_2_ with abundant oxygen vacancies for efficient acidic electrocatalytic hydrogen evolution. Angew. Chem. Int. Ed..

[21] Deng L (2021). Electronic modulation caused by interfacial Ni-O-M (M=Ru, Ir, Pd) bonding for accelerating hydrogen evolution kinetics. Angew. Chem. Int. Ed..

[22] Wang W, Wang Z, Hu Y, Liu Y, Chen S (2022). A potential-driven switch of activity promotion mode for the oxygen evolution reaction at Co_3_O_4_/NiO_x_H_y_ interface. eScience.

[23] Park JE (2022). Three-dimensional unified electrode design using a NiFeOOH catalyst for superior performance and durable anion-exchange membrane water electrolyzers. ACS Catal..

[24] Song W (2023). Upscaled production of an ultramicroporous anion-exchange membrane enables long-term operation in electrochemical energy devices. Nat. Commun..

[25] Wu Z-Y (2023). Non-iridium-based electrocatalyst for durable acidic oxygen evolution reaction in proton exchange membrane water electrolysis. Nat. Mater..

[26] Wang J (2021). Redirecting dynamic surface restructuring of a layered transition metal oxide catalyst for superior water oxidation. Nat. Catal..

[27] Kosmala T (2021). Operando visualization of the hydrogen evolution reaction with atomic-scale precision at different metal–graphene interfaces. Nat. Catal..

[28] Wang X (2022). Electronic structure modulation of RuO_2_ by TiO_2_ enriched with oxygen vacancies to boost acidic O_2_ evolution. ACS Catal..

[29] Lin C (2021). In-situ reconstructed Ru atom array on α-MnO_2_ with enhanced performance for acidic water oxidation. Nat. Catal..

[30] Shi X (2022). Efficient and stable acidic water oxidation enabled by low-concentration, high-valence iridium sites. ACS Energy Lett..

[31] Liu X (2021). Restructuring highly electron-deficient metal-metal oxides for boosting stability in acidic oxygen evolution reaction. Nat. Commun..

[32] Mahdavi-Shakib A (2023). The role of surface hydroxyls in the entropy-driven adsorption and spillover of H_2_ on Au/TiO_2_ catalysts. Nat. Catal..

[33] Han, H. G., Choi, J. W., Son, M. & Kim, K. C. Unlocking power of neighboring vacancies in boosting hydrogen evolution reactions on two-dimensional NiPS_3_ monolayer. *eScience*, 100204 10.1016/j.esci.2023.100204 (2023).

[34] Xia J (2022). Ti_n_O2_n–1_/MXene hierarchical bifunctional catalyst anchored on graphene aerogel toward flexible and high-energy Li–S batteries. ACS Nano.

[35] Yang B (2023). Flatband λ-Ti_3_O_5_ towards extraordinary solar steam generation. Nature.

[36] Dai J (2020). Single-phase perovskite oxide with super-exchange induced atomic-scale synergistic active centers enables ultrafast hydrogen evolution. Nat. Commun..

[37] Ioroi T, Siroma Z (2019). Yamazaki S.-i. & Yasuda K. Electrocatalysts for PEM fuel cells. Adv. Energy Mater..

[38] Hao Y (2023). Switching the oxygen evolution mechanism on atomically dispersed Ru for enhanced acidic reaction kinetics. J. Am. Chem. Soc..

[39] He Q (2022). Confining high-valence iridium single sites onto nickel oxyhydroxide for robust oxygen evolution. Nano Lett..

[40] Hu F (2022). Lattice-matching formed mesoporous transition metal oxide heterostructures advance water splitting by active Fe–O–Cu bridges. Adv. Energy Mater..

[41] Zhang B (2023). Enhanced spatial charge separation in a niobium and tantalum nitride core-shell photoanode: In situ interface bonding for efficient solar water splitting. Angew. Chem. Int. Ed..

[42] Chen R (2023). Promoting the efficiency and selectivity of NO_3_^−^−to−NH_3_ reduction on Cu−O−Ti active sites via preferential glycol oxidation with holes. P. Natl Acad. Sci. USA.

[43] Li M (2021). Revealing the regulation mechanism of Ir–MoO_2_ interfacial chemical bonding for improving hydrogen oxidation reaction. ACS Catal..

[44] Dai J (2022). Hydrogen spillover in complex oxide multifunctional sites improves acidic hydrogen evolution electrocatalysis. Nat. Commun..

[45] Fu HQ (2022). Hydrogen spillover-bridged volmer/tafel processes enabling ampere-Level current density alkaline Hydrogen evolution reaction under low overpotential. J. Am. Chem. Soc..

[46] Zheng T (2019). Intercalated iridium diselenide electrocatalysts for efficient pH-universal water splitting. Angew. Chem. Int. Ed..

[47] Li J (2021). A fundamental viewpoint on the hydrogen spillover phenomenon of electrocatalytic hydrogen evolution. Nat. Commun..

[48] Hou L (2023). Electronic and lattice engineering of ruthenium oxide towards highly active and stable water splitting. Adv. Energy Mater..

[49] Nong HN (2020). Key role of chemistry versus bias in electrocatalytic oxygen evolution. Nature.

[50] Yang L (2023). A highly active, long-lived oxygen evolution electrocatalyst derived from open-framework iridates. Adv. Mater..

[51] Hao Y (2023). Switchingsed Ru for enhanced acidic reaction kinetics. J. Am. Chem. Soc..

[52] Du K (2022). Interface engineering breaks both stability and activity limits of RuO_2_ for sustainable water oxidation. Nat. Commun..

[53] Yao Y (2019). Engineering the electronic structure of single atom Ru sites via compressive strain boosts acidic water oxidation electrocatalysis. Nat. Catal..

[54] Zhang Q (2019). Solid-solution alloy nanoparticles of a combination of immiscible Au and Ru with a large gap of reduction potential and their enhanced oxygen evolution reaction performance. Chem. Sci..

[55] Yang Y (2019). Hierarchical nano assembly of MoS_2_/Co_9_S_8_/Ni_3_S_2_/Ni as a highly efficient electrocatalyst for overall water splitting in a wide pH range. J. Am. Chem. Soc..

[56] Zheng X (2022). Enriched d-band holes enabling fast oxygen evolution kinetics on atomic-layered defect-rich lithium cobalt oxide nanosheets. Adv. Funct. Mater..

[57] Tan H (2022). Engineering a local acid-like environment in alkaline medium for efficient hydrogen evolution reaction. Nat. Commun..

[58] Hammer B, Norskov JK (1995). Why gold is the noblest of all the metals. Nature.

[59] Ruban A, Hammer B, Stoltze P, Skriver HL, Nørskov JK (1997). Surface electronic structure and reactivity of transition and noble metals. J. Mol. Catal. A: Chem..

[60] Bornet A (2023). Influence of temperature on the performance of carbon- and ATO-supported oxygen evolution reaction catalysts in a gas diffusion electrode setup. ACS Catal..

[61] Kwon J (2023). Tailored electronic structure of Ir in high entropy alloy for highly active and durable bifunctional electrocatalyst for water splitting under an acidic environment. Adv. Mater..

[62] Blöchl PE (1994). Projector augmented-wave method. Phys. Rev. B.

[63] Perdew JP, Burke K, Ernzerhof M (1996). Generalized gradient approximation made simple. Phys. Rev. Lett..

[64] Hjorth Larsen A (2017). The atomic simulation environment—a Python library for working with atoms. J. Phys.: Condens. Matter.

