# Supplementary material for: Constructing regulable supports via non-stoichiometric engineering to stabilize ruthenium nanoparticles for enhanced pH-universal water splitting
Source: Nat Commun. 2024 Mar 29;15:2728. doi: 10.1038/s41467-024-46750-6 (PMC10980754; doi:10.1038/s41467-024-46750-6)
Supplement: Supplementary file 1 — Supplementary Information [file 41467_2024_46750_MOESM1_ESM.pdf]

Supplementary information

Constructing regulable supports via non-stoichiometric engineering to stabilize ruthenium nanoparticles for enhanced pH-universal water splitting

Sheng Zhao[1], Sung-Fu Hung[2], Liming Deng[1], Wen-Jing Zeng[2], Tian Xiao[1], Shaoxiong Li[1], Chun-Han Kuo[3], Han-Yi Chen[3], Feng Hu[1], and Shengjie Peng[1]*

[1]College of Materials Science and Technology, Nanjing University of Aeronautics and Astronautics, Nanjing 210016, China.

[2]Department of Applied Chemistry, National Yang Ming Chiao Tung University, Hsinchu 300, Taiwan.

[3]Department of Materials Science and Engineering, National Tsing Hua University 101, Sec. 2, Kuang-Fu Road, Hsinchu 30013, Taiwan.

*Corresponding author.

Email: pengshengjie@nuaa.edu.cn

1 Supplementary Figures

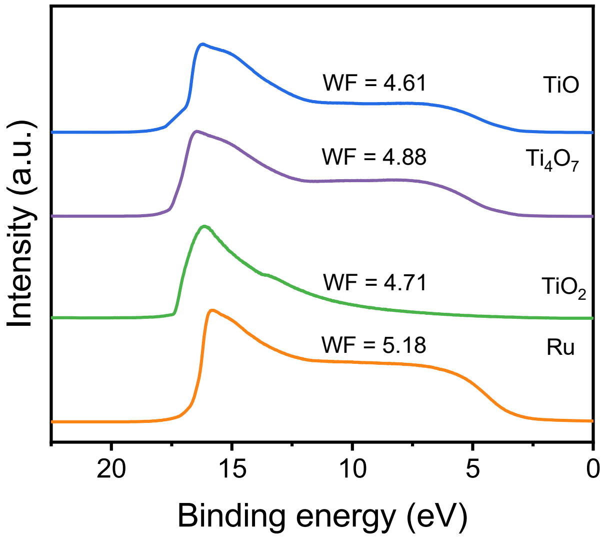

Supplementary Fig. 1. Valence band structure characterization. Ultraviolet photoelectron spectroscopy (UPS) of Ru, TiO_2, Ti_4O_7, and TiO, respectively.

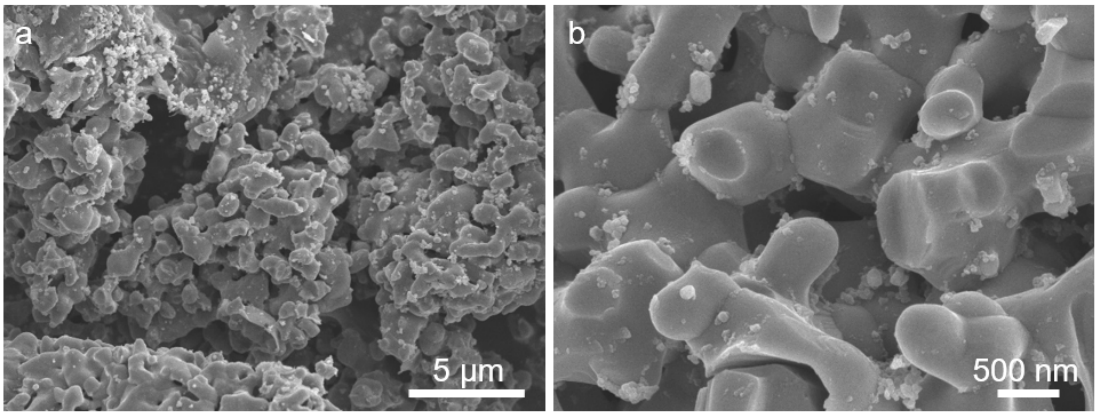

Supplementary Fig. 2. Morphology characterization. a Low magnified and **b** high magnified SEM images of Ti_4O_7, respectively.

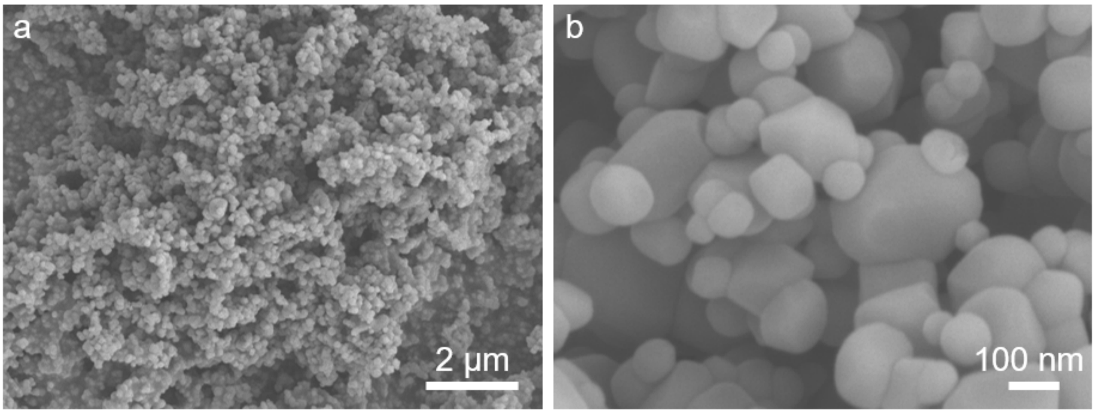

Supplementary Fig. 3. Morphology characterization. **a** Low magnified, and **b** high magnified SEM images of TiO_2, respectively.

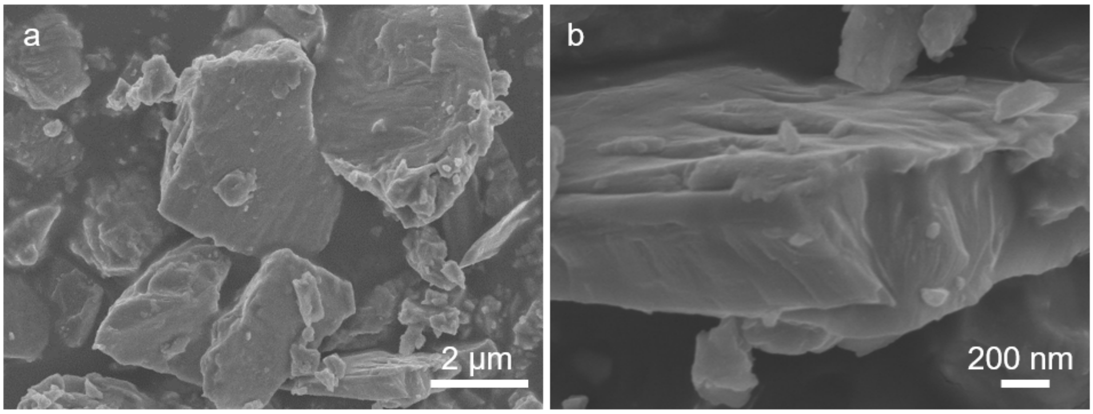

Supplementary Fig. 4. Morphology characterization. **a** Low magnified, and **b** high magnified SEM images of TiO, respectively.

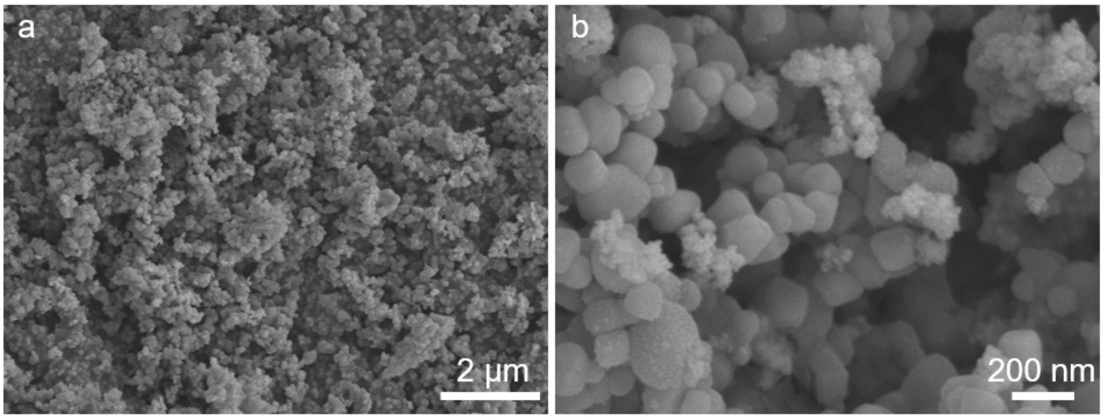

Supplementary Fig. 5. Morphology characterization. a Low magnified and **b** high magnified SEM images of Ru/TiO_2, respectively.

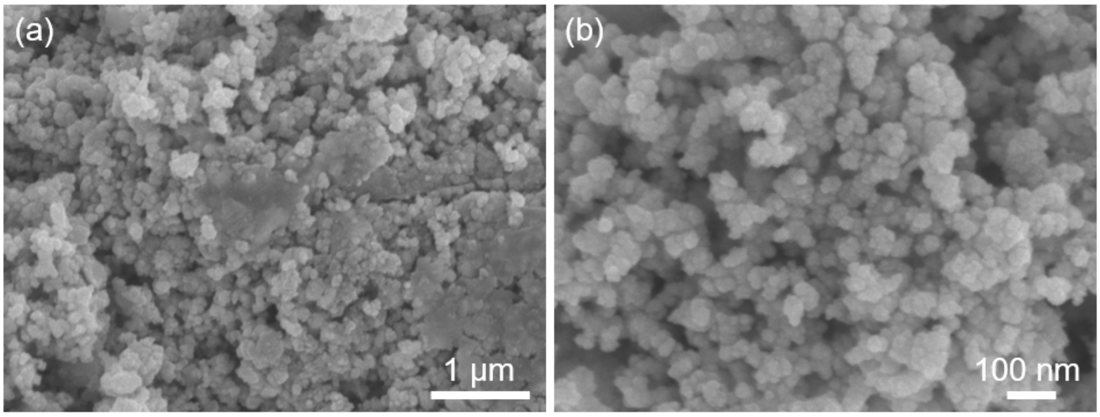

Supplementary Fig. 6. Morphology characterization. a Low magnified and **b** high magnified SEM images of Ru/TiO, respectively.

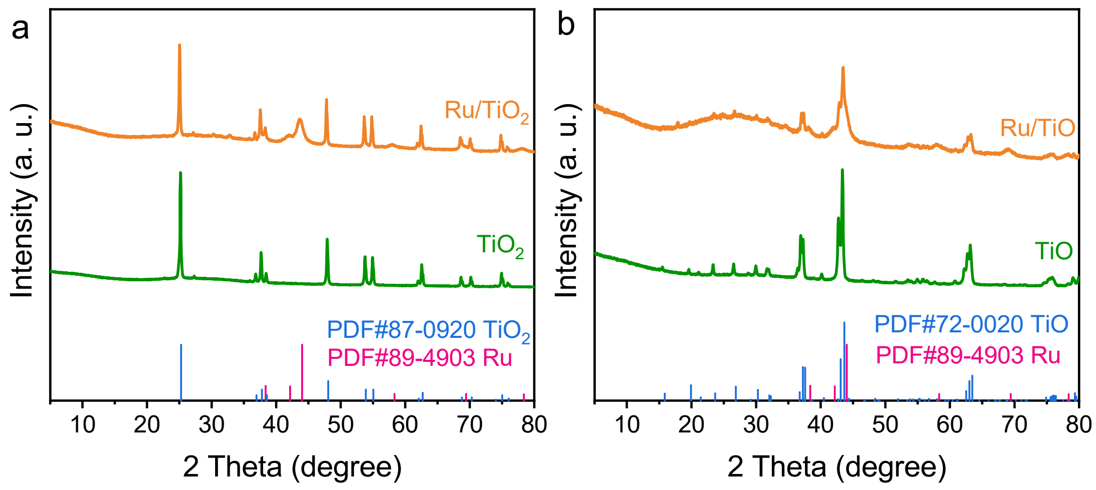

Supplementary Fig. 7. Phase characterization. a XRD patterns of TiO_2, and Ru/TiO_2, respectively. **b** XRD patterns of TiO and Ru/TiO, respectively.

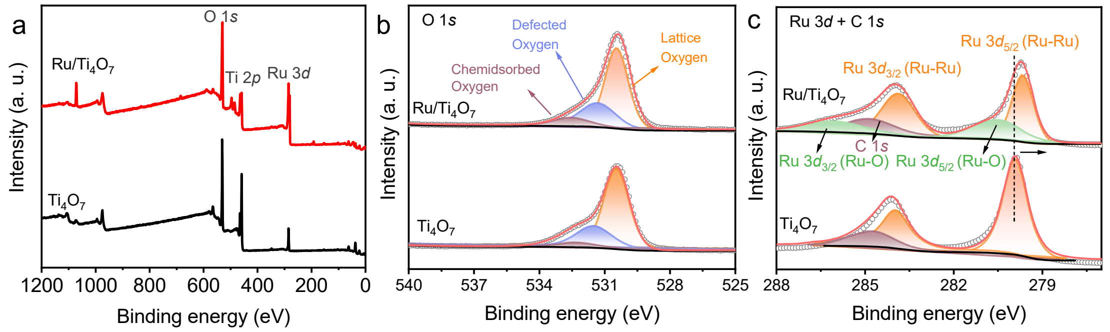

Supplementary Fig. 8. XPS tests. a XPS survey spectra of Ti_4O_7, and Ru/Ti_4O_7. **b** XPS fine spectra of O 1*s* of Ti_4O_7, and Ru/Ti_4O_7, respectively. **c** XPS fine spectra of Ru 3*d* and C 1*s* of Ru, and Ru/Ti_4O_7, respectively.

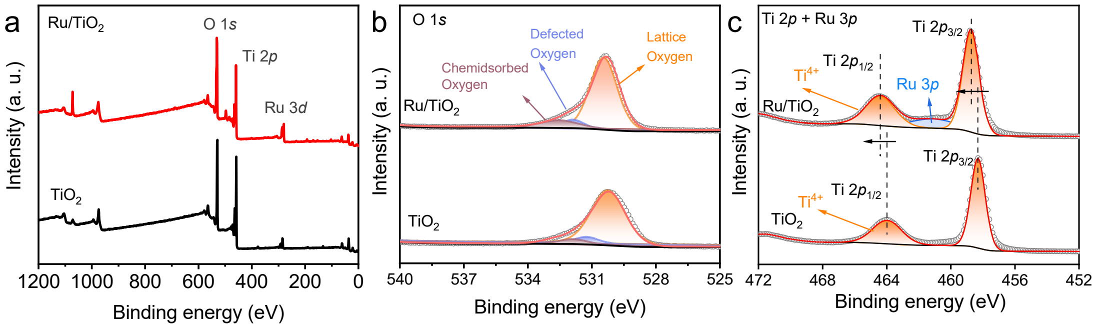

Supplementary Fig. 9. XPS tests. a XPS survey spectra of TiO_2, and Ru/TiO_2. XPS fine spectra of **b** O 1*s* and **c** Ti 2*p* with Ru 3*p* of TiO_2 and Ru/TiO_2, respectively.

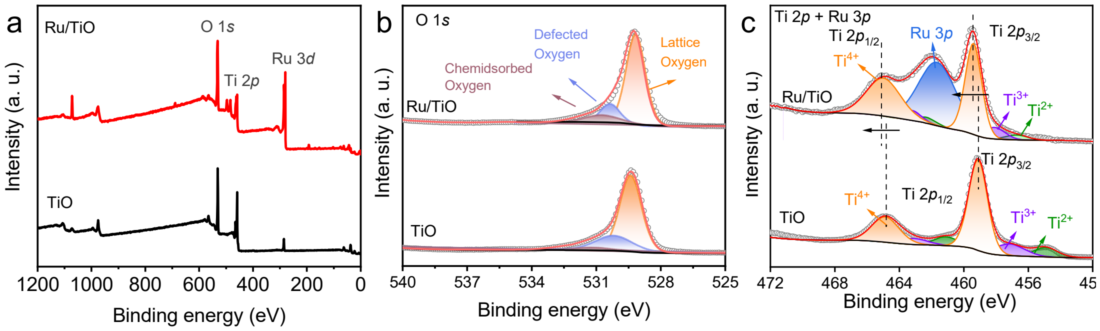

Supplementary Fig. 10. XPS tests. a XPS survey spectra of TiO, and Ru/TiO. XPS fine spectra of **b** O 1*s* and **c** Ti 2*p* with Ru 3*p* of TiO and Ru/TiO, respectively.

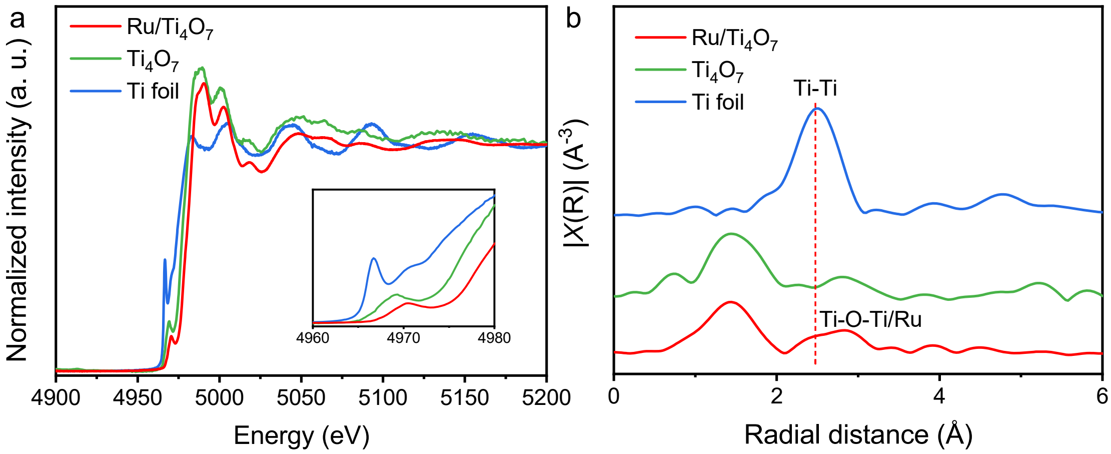

Supplementary Fig. 11. Electronic structure characterization. a Normalized Ti *K*-edge XANES of Ti foil, Ru/TiO_2, Ru/Ti_4O_7, and Ru/TiO. **b** The corresponding k^2-weighted Fourier transforms.

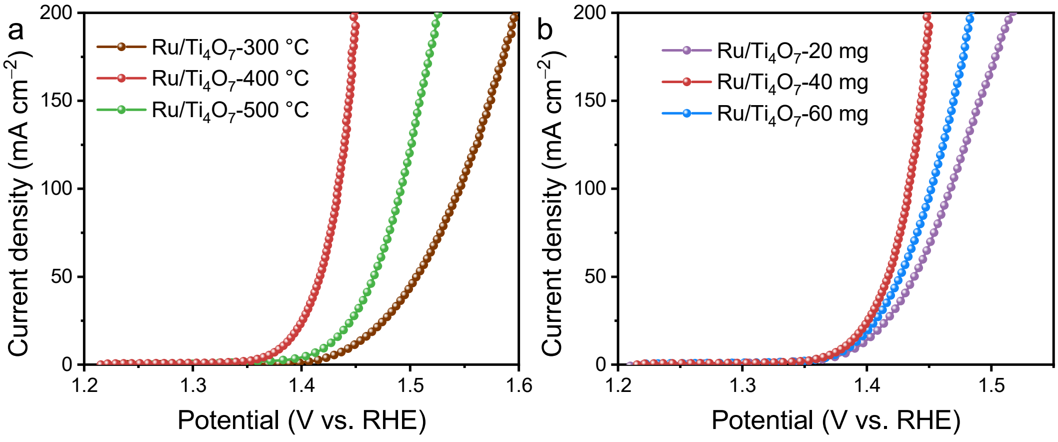

Supplementary Fig. 12. Activity optimization. OER LSV polarization curves of **a** Ru/Ti_4O_7 prepared at different temperatures, and **b** Ru/Ti_4O_7 prepared using $RuCl_3$ precursors.

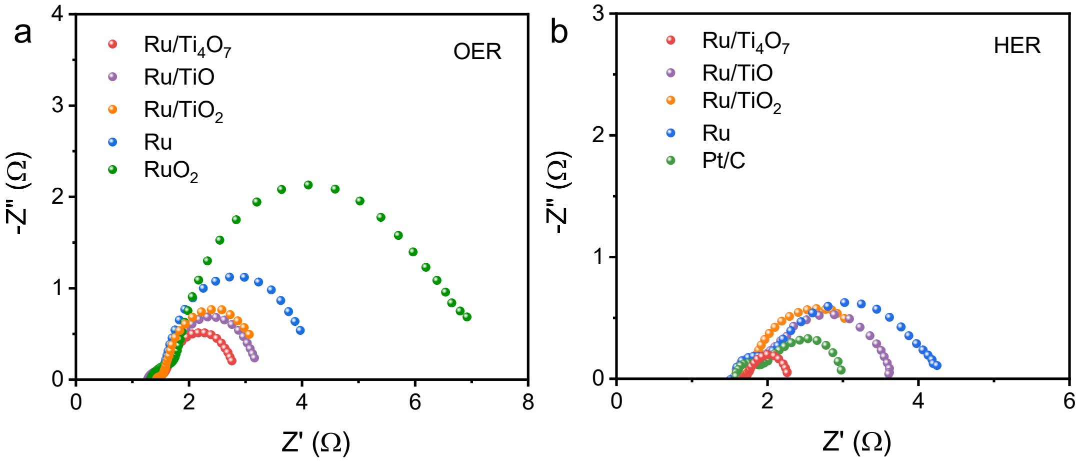

Supplementary Fig. 13. Electrochemical impedance spectroscopy (EIS) tests. a EIS Nyquist curves of Ru, commercial RuO_2, Ru/TiO_2, Ru/Ti_4O_7, and Ru/TiO for OER in 0.5 M H_2SO_4. **b** The corresponding EIS Nyquist curves for HER in 0.5 M H_2SO_4.

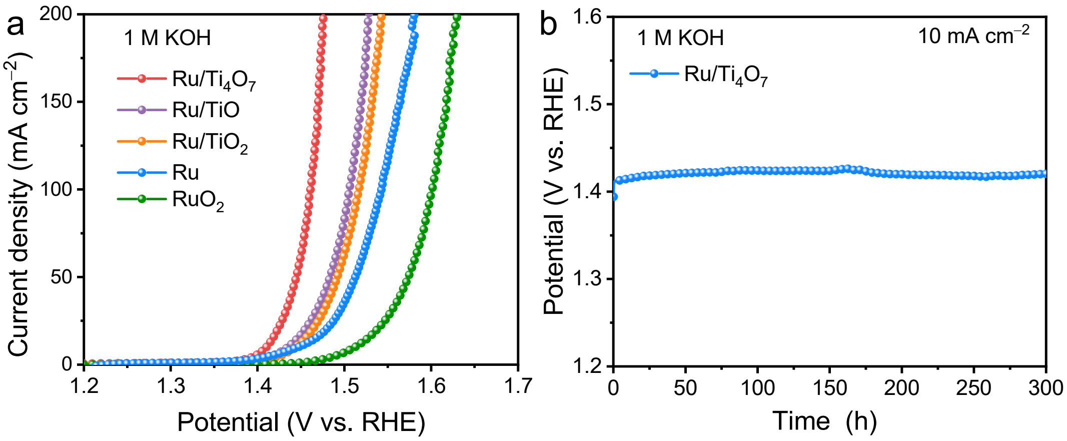

Supplementary Fig. 14. OER activity and stability characterization in the alkaline environment. a OER LSV polarization curves of Ru, commercial RuO_2, Ru/TiO_2, Ru/Ti_4O_7, and Ru/TiO in 1 M KOH. **b** Chronopotentiometry tests of Ru/Ti_4O_7 at 10 mA cm^{-2} in 1 M KOH.

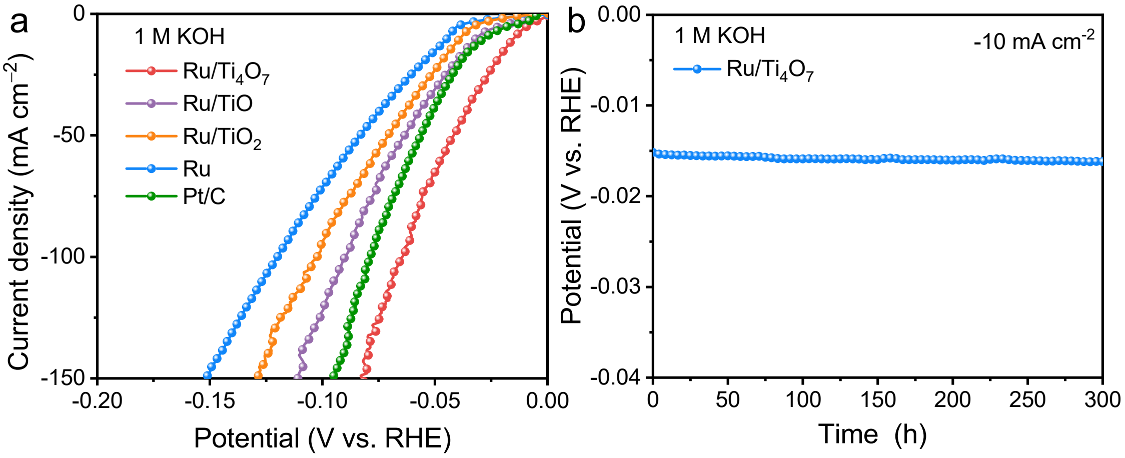

Supplementary Fig. 15. HER activity and stability characterization in the alkaline environment. a HER LSV polarization curves of Ru, commercial RuO_2, Ru/TiO_2, Ru/Ti_4O_7, and Ru/TiO in 1 M KOH. **b** Chronopotentiometry tests of Ru/Ti_4O_7 at −10 mA cm^{-2} in 1 M KOH.

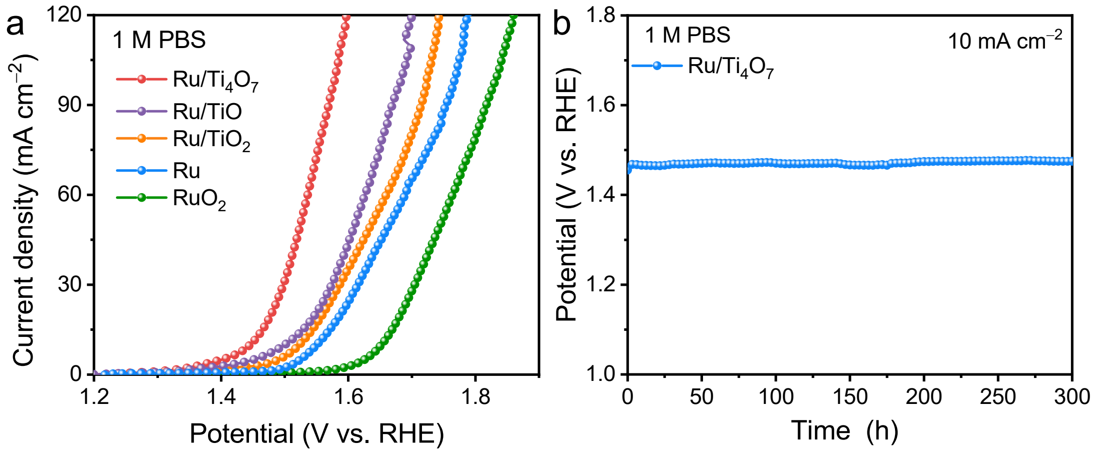

Supplementary Fig. 16. OER activity and stability characterization in the neutral environment. a OER LSV polarization curves of Ru, commercial RuO_2, Ru/TiO_2, Ru/Ti_4O_7, and Ru/TiO in 1 M PBS. **b** Chronopotentiometry tests of Ru/Ti_4O_7 at 10 mA cm^{-2} in 1 M PBS.

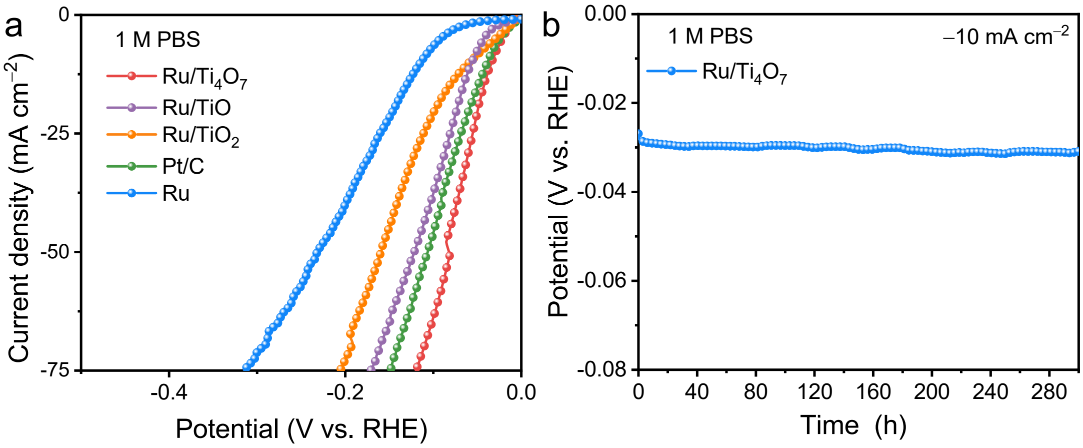

Supplementary Fig. 17. HER activity and stability characterization in the neutral environment. a HER LSV polarization curves of Ru, commercial RuO_2, Ru/TiO_2, Ru/Ti_4O_7, and Ru/TiO in 1 MPBS. **b** Chronopotentiometry tests of Ru/Ti_4O_7 at −10 mA cm^{-2} in 1 M PBS.

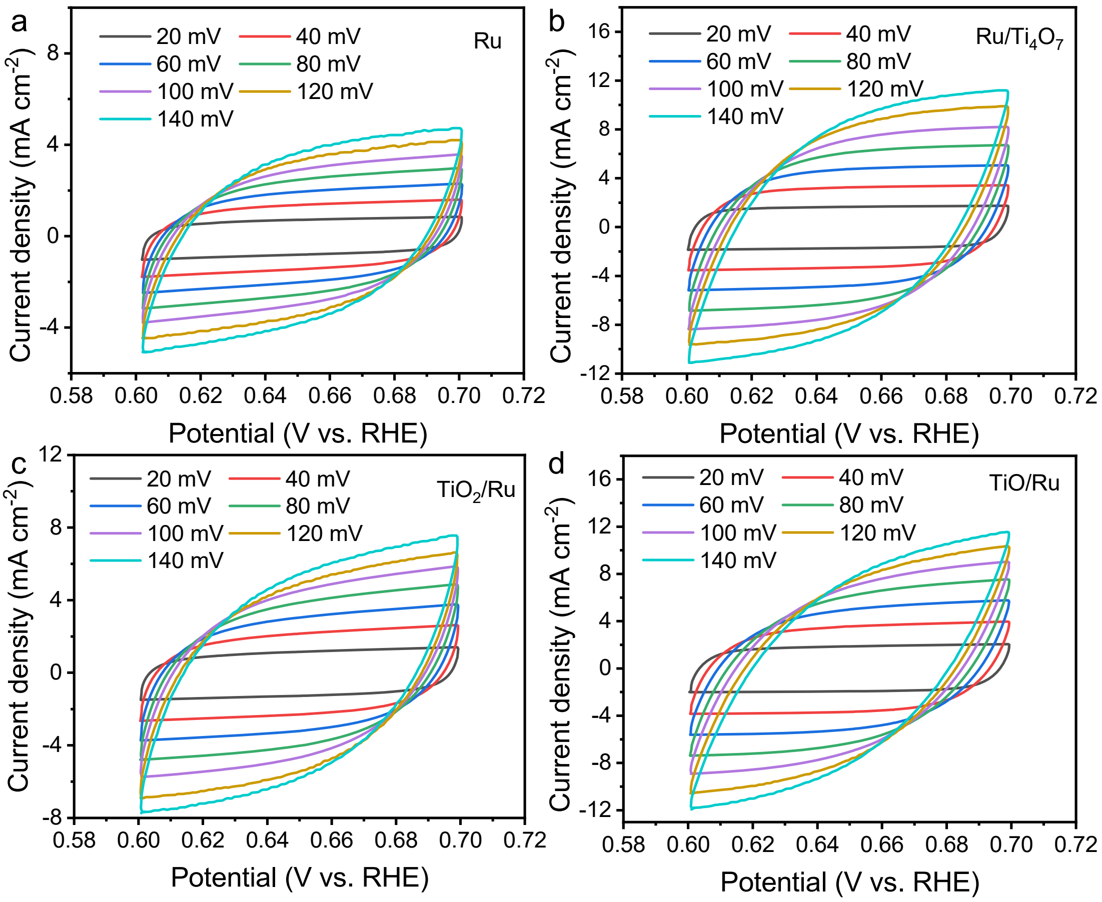

Supplementary Fig. 18. Double-layer capacitance tests. Cyclic voltammograms (CV) curves of **a** Ru, **b** Ru/Ti_4O_7, **c** Ru/TiO_2, and **d** Ru/TiO at various scan rates (20~140 mV s^{-1}) under the potential range of 0.6-0.7 V vs. RHE, which were used to estimate double-layer capacitance (C_{dl}).

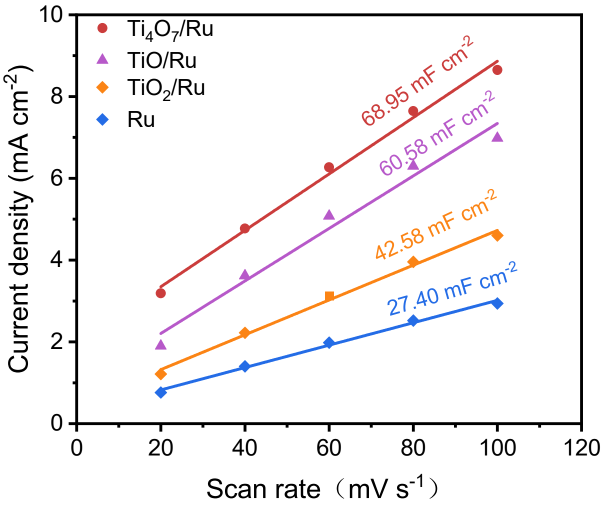

Supplementary Fig. 19. Double-layer capacitance evaluation. Linear fitting of the current density versus scan rates of Ru, Ni-Ru/TiO_2, Ru/Ti_4O_7, and Ru/TiO, and the corresponding value of C_{dl}.

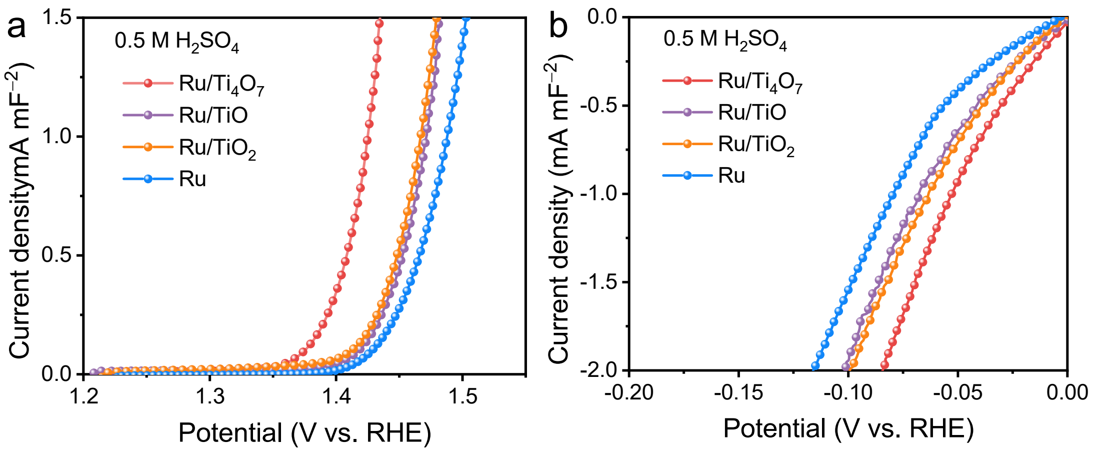

Supplementary Fig. 20. OER specific activity evaluation. C_{dl} normalized LSV polarization curves of different catalysts for **a** OER and **b** HER, respectively.

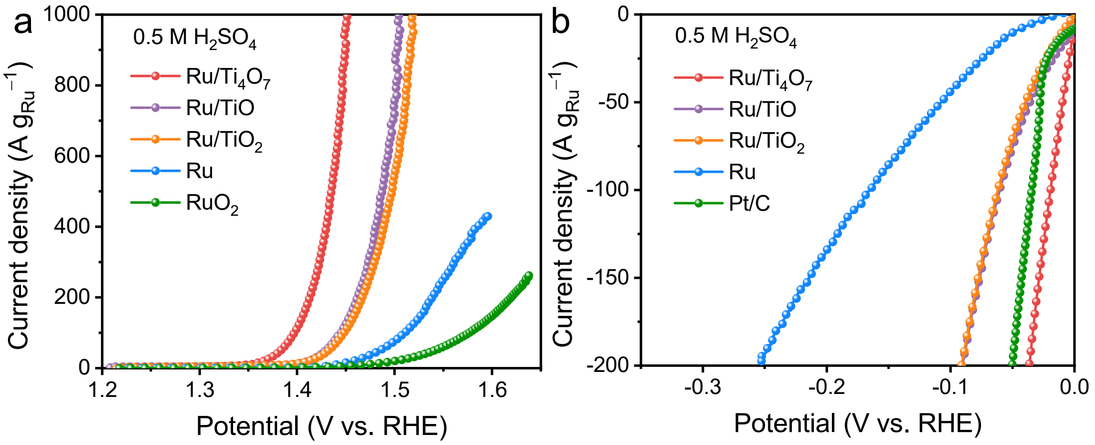

Supplementary Fig. 21. OER mass activity evaluation. Mass activity of different catalysts for **a** OER and **b** HER, respectively.

The Ru content was confirmed via inductively coupled plasma-mass spectrometry (ICP-MS), as shown in Supplementary Table 1, to calculate the mass activity of different catalysts.

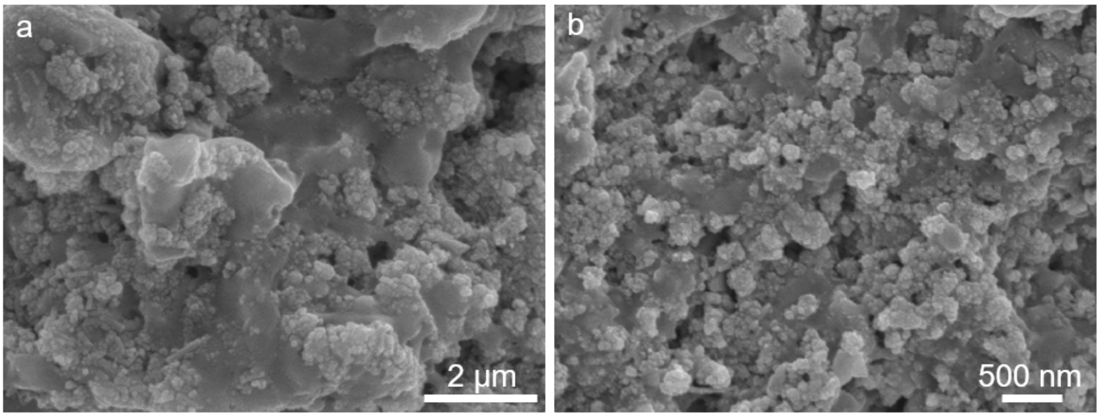

Supplementary Fig. 22. Morphology characterization after OER. a Low magnified and **b** high magnified SEM images of Ru/Ti_4O_7 after OER cycling, respectively.

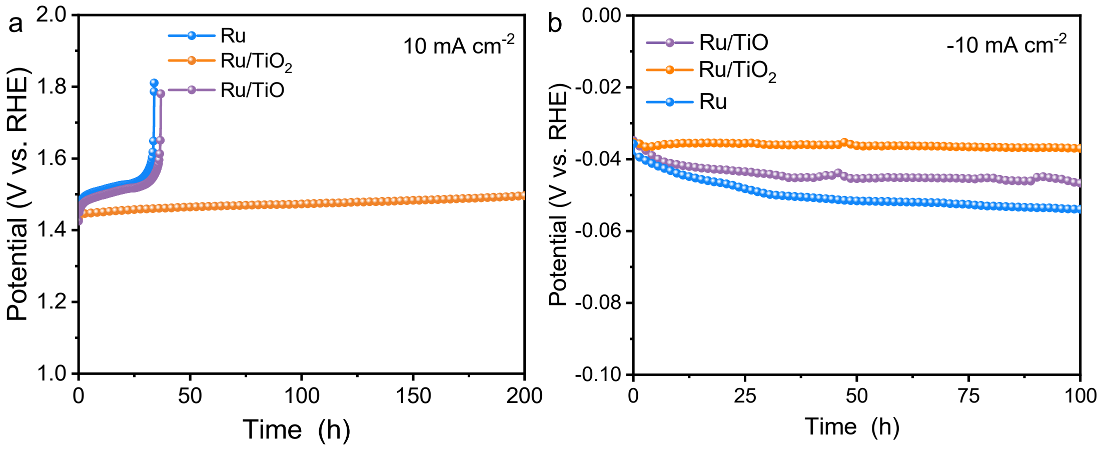

Supplementary Fig. 23. Stability tests of other material in the acidic environment. Chronopotentiometry tests of Ru Ru/TiO_2 and Ru/TiO at **a** 10 mA cm^{-2} **b** −10 mA cm^{-2} in 0.5 M H_2SO_4.

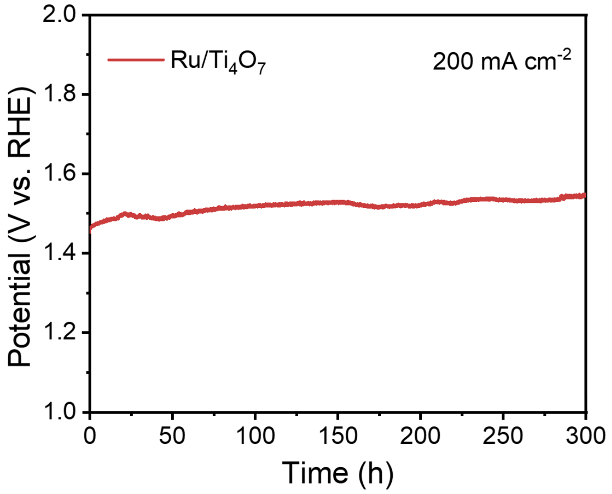

Supplementary Fig. 24. Stability test under high-current-density in the acidic environment.

Chronopotentiometry curves of Ru/Ti_4O_7 at 200 mA cm^{-2} in 0.5 M H_2SO_4.

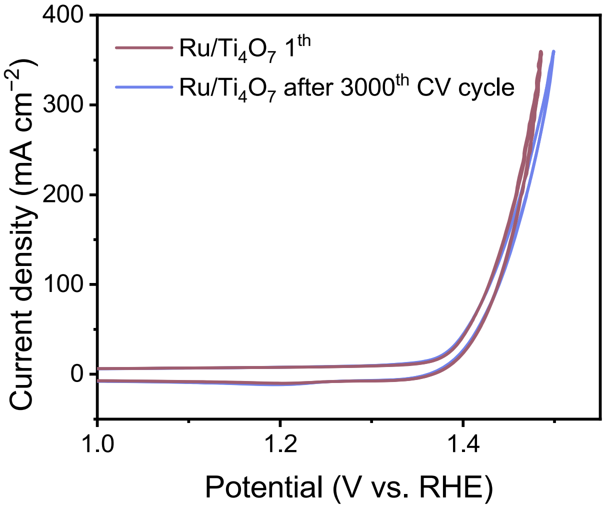

Supplementary Fig. 25. Stability in the acidic environment. OER CV curves of Ru/Ti_4O_7 before and after 3000th cycles.

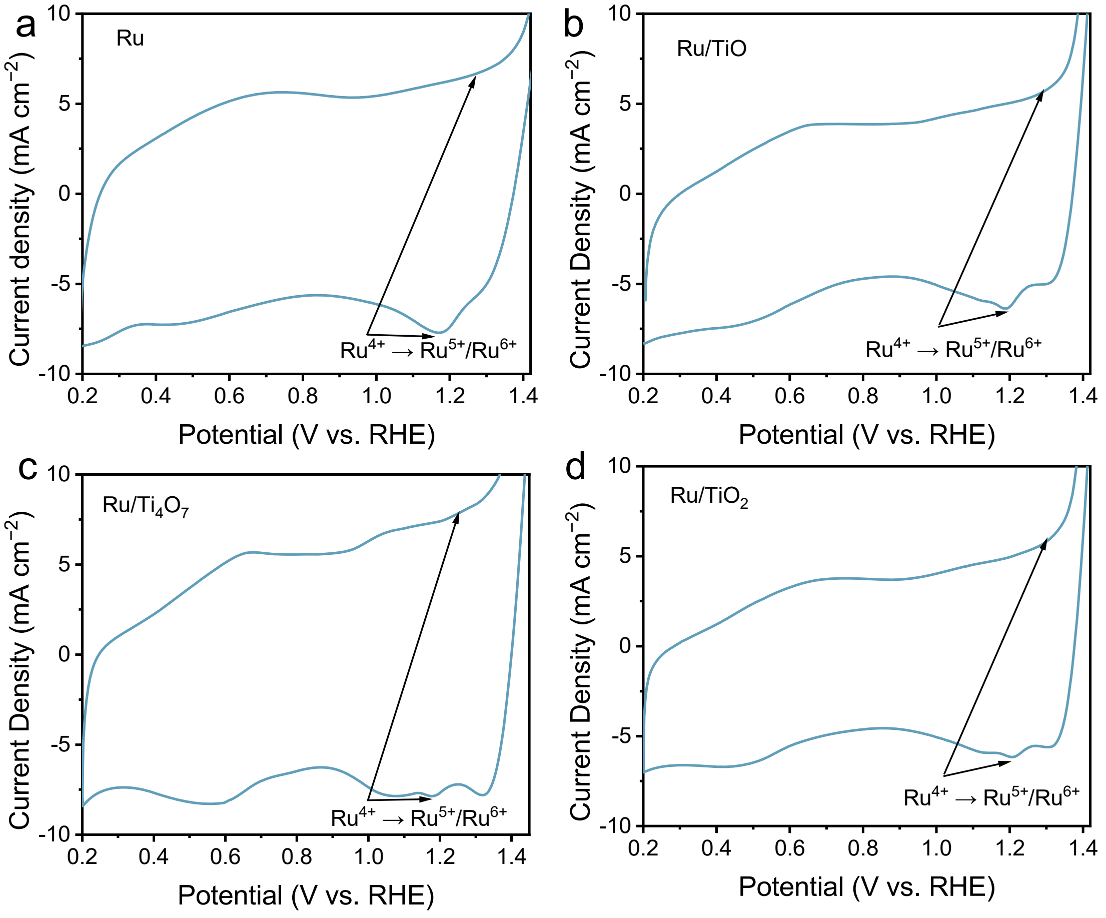

Supplementary Fig. 26. Electrochemical reconstruction process of different materials. CV curves of **a-d** Ru, Ru/TiO, Ru/Ti4O7, and Ru/TiO2, respectively, without iR-corrected in argon-saturated 0.5 M H_2SO_4 at 50 mV s^{-1} without iR compensation.

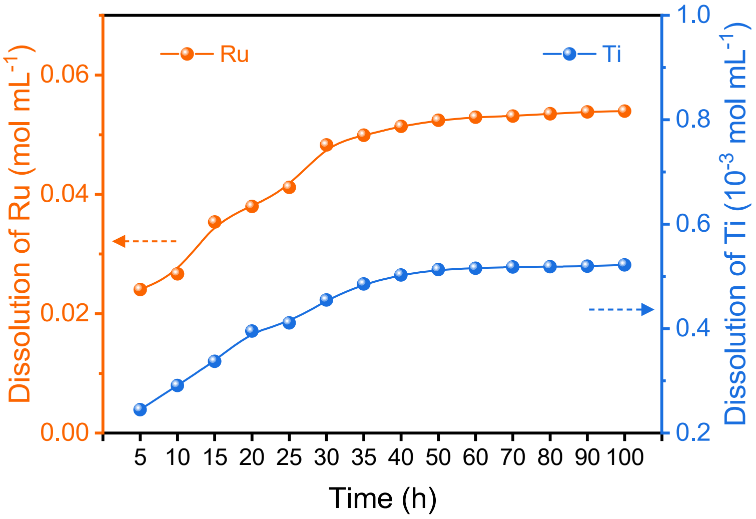

Supplementary Fig. 27. Material dissolution detection during OER. Dissolved Ru (left ordinate - y-axis) and Ti (right ordinate - y-axis) ion concentration in electrolyte for Ru/Ti_4O_7 determined via ICP-MS.

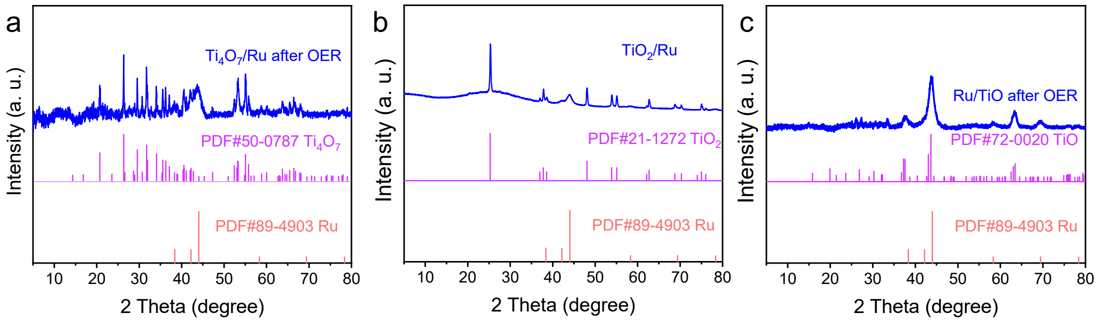

Supplementary Fig. 28. Phase characterization after OER. XRD patterns of **a** Ru/Ti_4O_7 after OER cycling, **b** Ru/TiO_2 after OER, and **c** Ru/TiO after OER, respectively.

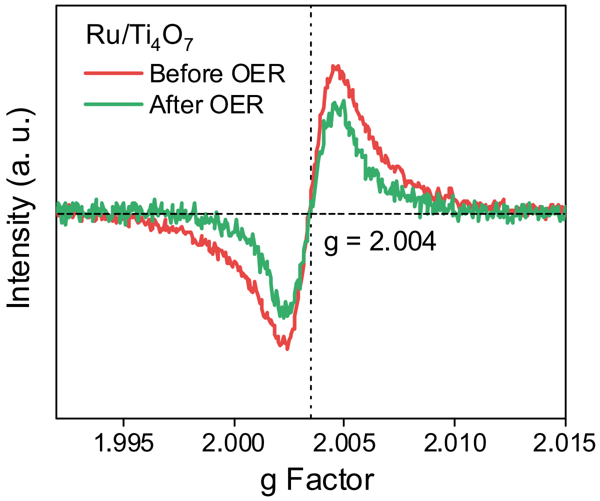

Supplementary Fig. 29. Defect degree change during OER. EPR spectra of Ru/Ti_4O_7 before and after the OER stability test at 200 mA cm^{-2}.

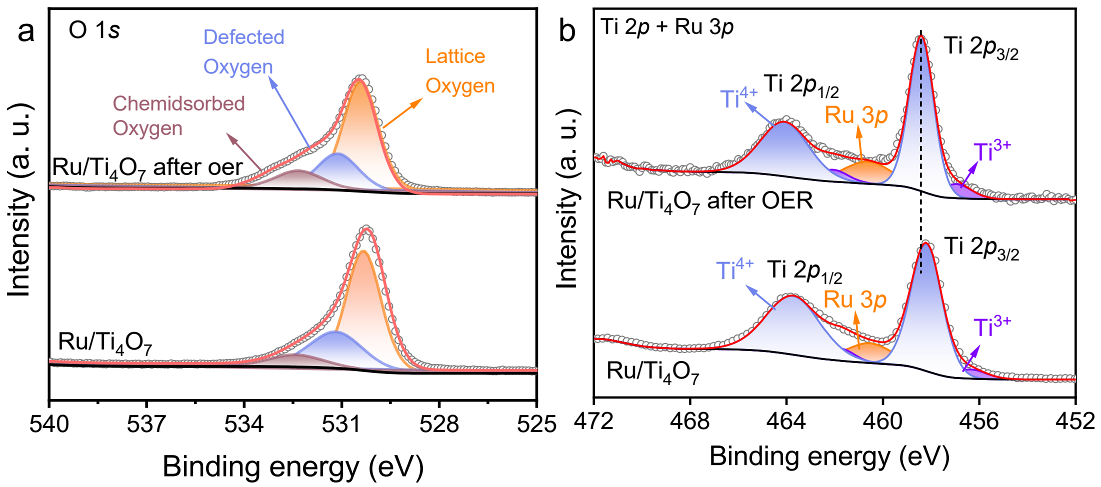

Supplementary Fig. 30. XPS tests after OER. XPS fine spectra of **a** O 1*s* and **b** Ti 2*p* with Ru 3*p* for Ru/Ti_4O_7 after OER.

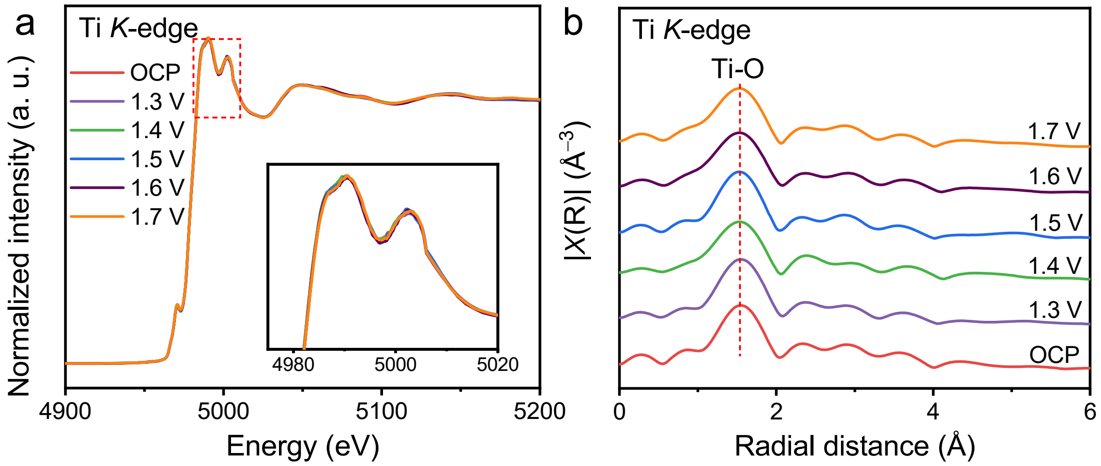

Supplementary Fig. 31. In-situ XANES and Fourier transforms k^2-weighted EXAFS spectra. **a** Normalized in-situ Ti *K*-edge XANES of Ru/Ti_4O_7. **b** The corresponding k^2-weighted Fourier transforms.

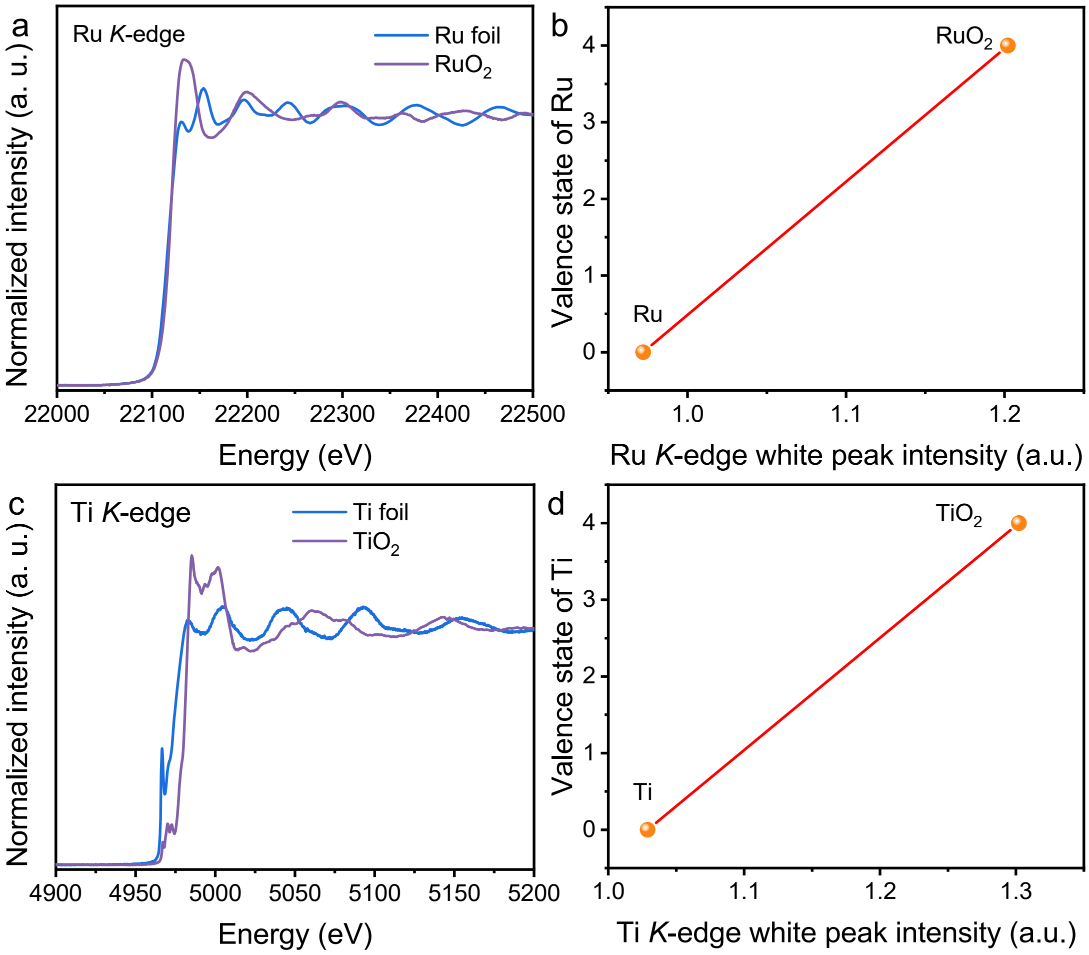

Supplementary Fig. 32. XAS chemical valence calibration. **a** Ru *K*-edge of Ru and RuO_2. **b** Calibration of Ru chemical valence against the relative edge white peak intensity deduced from the reference materials. **c** Ru *K*-edge of Ru and TiO_2. **d** Calibration of Ti chemical valence against the relative edge white peak intensity deduced from the reference materials.

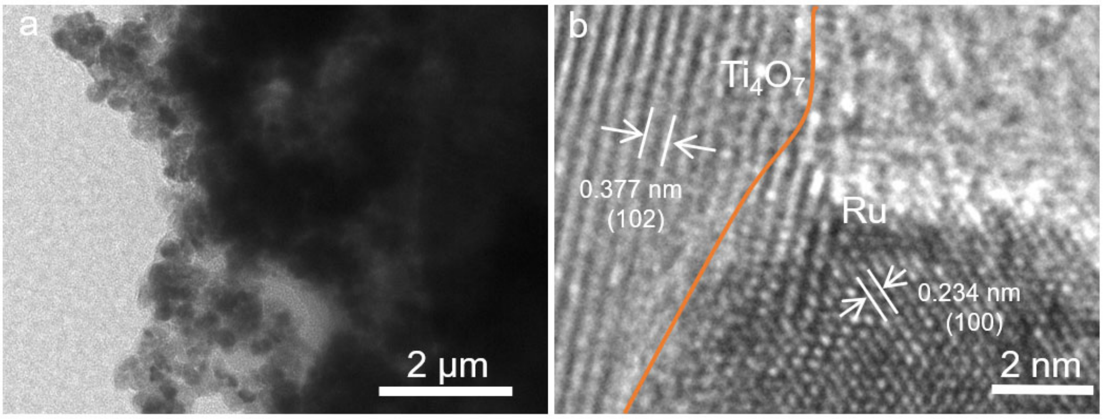

Supplementary Fig. 33. TEM characterization. **a** TEM and **b** HRTEM images of Ru/Ti_4O_7 after OER cycling, respectively.

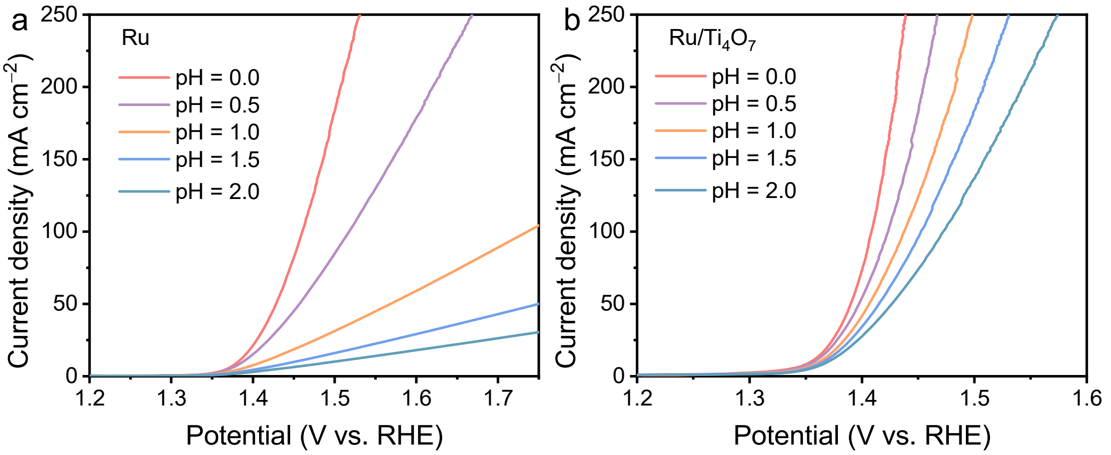

Supplementary Fig. 34. pH dependence tests. LSV of **a** Ru and **b** Ru/Ti_4O_7 measured in different pH environments without iR compensation.

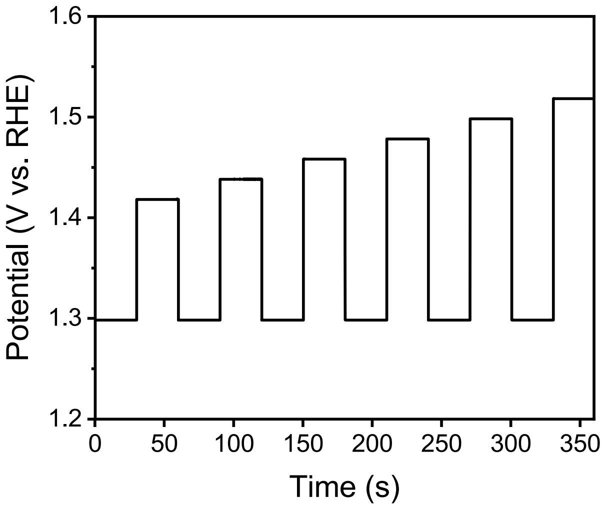

Supplementary Fig. 35. Pulse voltammetry tests. Pulse voltammetry protocol between 1.35 V cathodic and 1.42 V to 1.50 V vs. RHE anodic potentials without iR compensation.

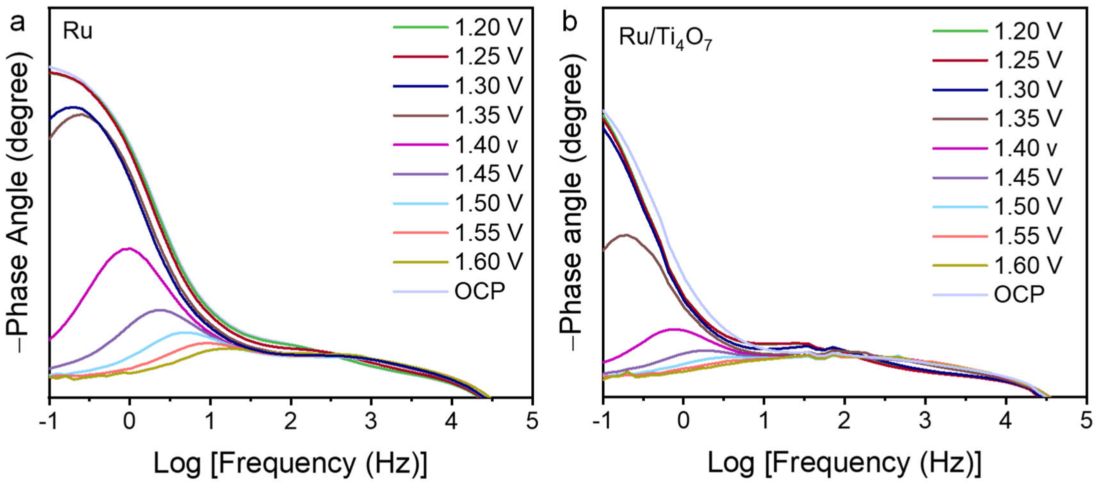

Supplementary Fig. 36. EIS tests to monitor the OER process. EIS Bode plots of **a** Ru and **b** Ru/Ti_4O_7 at the potentials of 1.20-1.60 V vs. RHE.

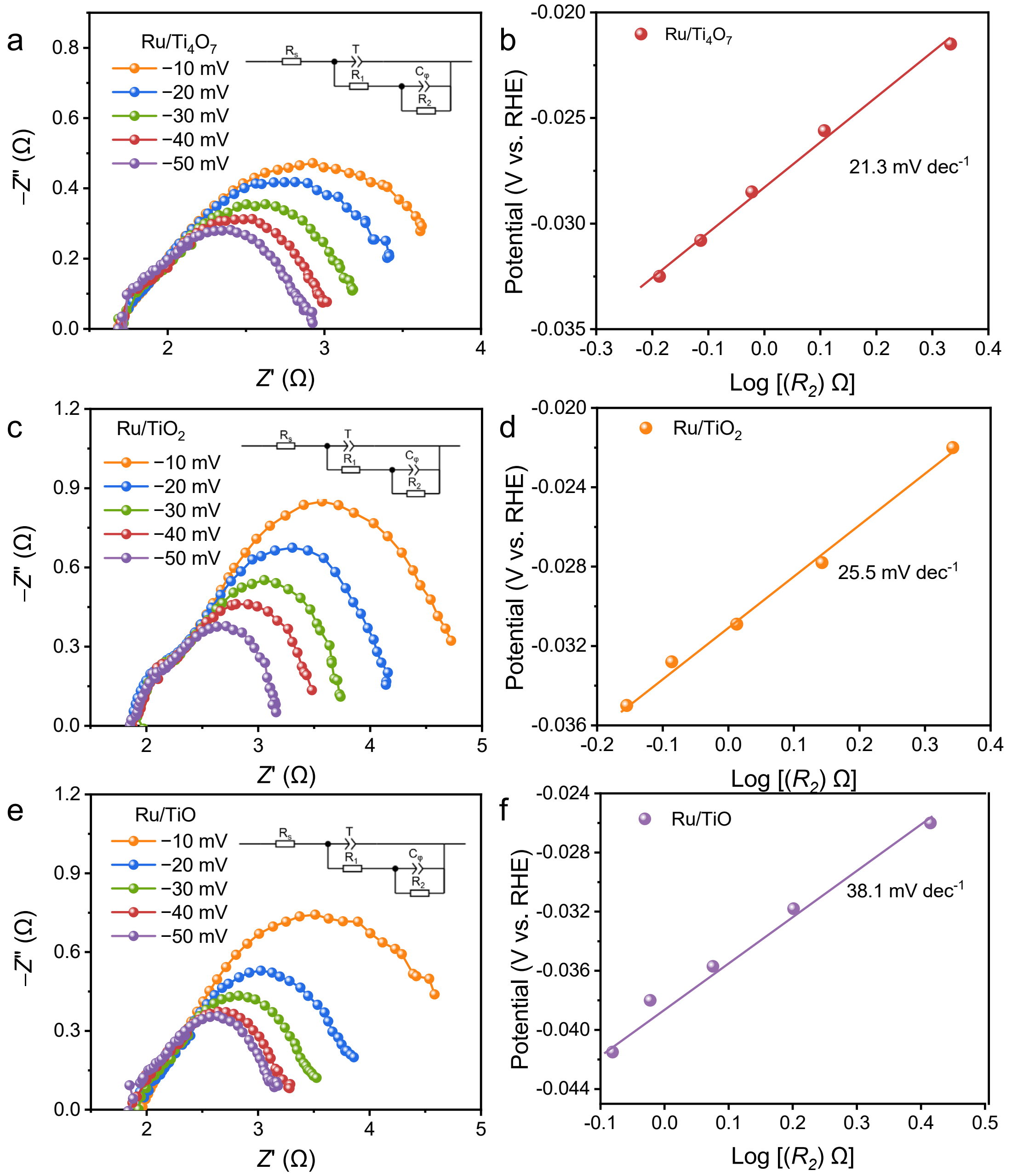

Supplementary Fig. 37. EIS tests to monitor the HER process. a EIS Nyquist curves of Ru/Ti_4O_7 in 0.5 M H_2SO_4 at various overpotentials. **b** EIS-derived Tafel plots of Ru/Ti_4O_7 obtained from the hydrogen adsorption resistance. **c** EIS Nyquist curves of Ru/TiO_2 in 0.5 M H_2SO_4 at various overpotentials. **d** EIS-derived Tafel plots of Ru/TiO_2 obtained from the hydrogen adsorption resistance. **e** EIS Nyquist curves of Ru/TiO in 0.5 M H_2SO_4 at various overpotentials. **f** EIS-derived Tafel plots of Ru/TiO obtained from the hydrogen adsorption resistance.

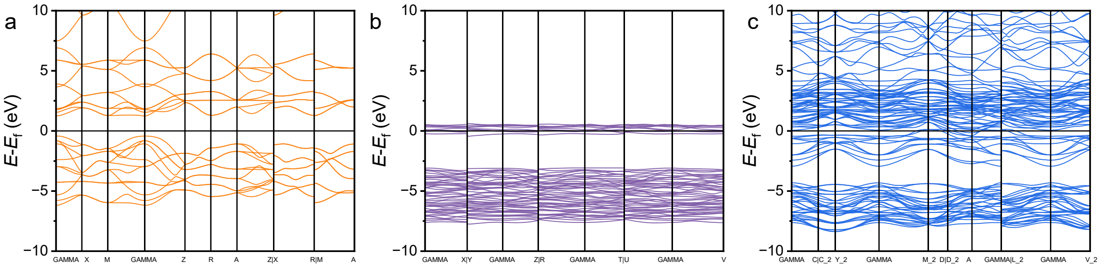

Supplementary Fig. 38. Energy band structure calculation. The energy band structures of **a** TiO_2, **b** Ti_4O_7, and **c** TiO, respectively.

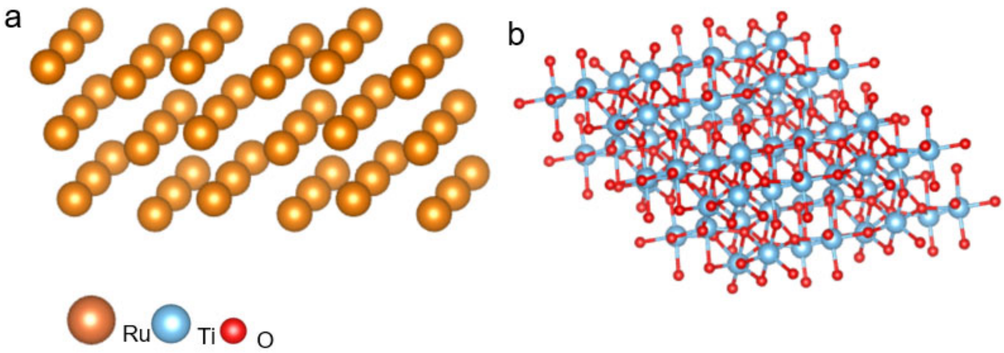

Supplementary Fig. 39. DFT calculated structural models. Relaxation structure of **a** Ru and **b** Ti_4O_7, respectively.

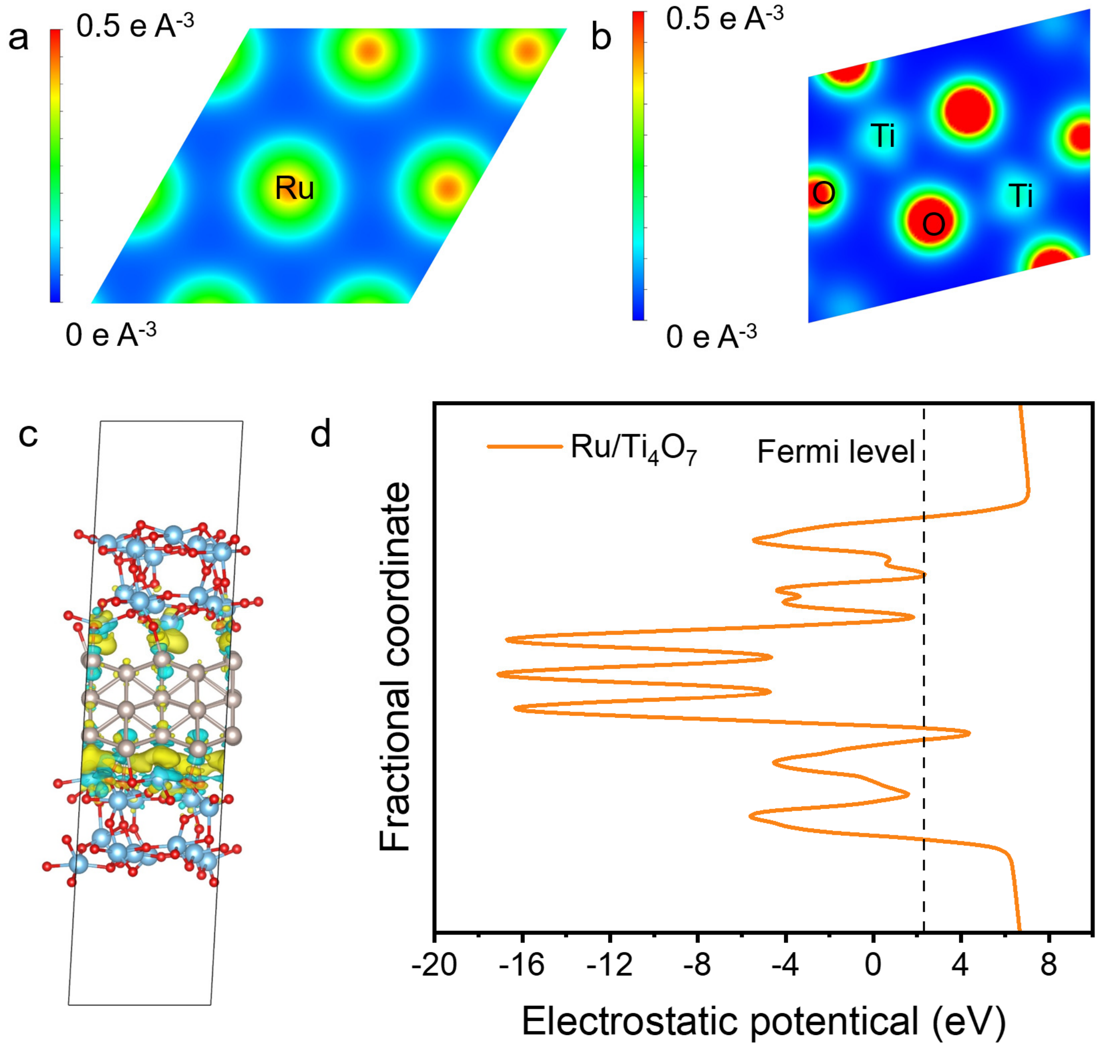

Supplementary Fig. 40. Calculation of the interfacial charge distribution. Charge density distribution of **a** Ru and **b** Ti_4O_7, respectively. **c** Charge density difference of Ru/Ti_4O_7. **d** Plane-average electron difference diagram of the interface between Ru and Ti_4O_7.

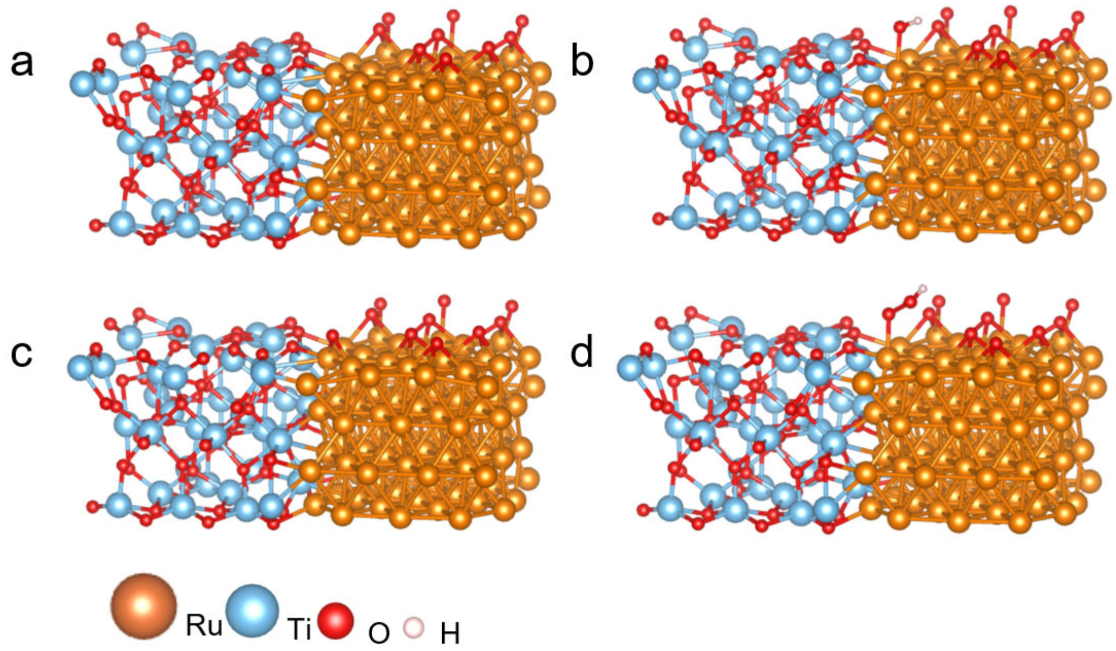

Supplementary Fig. 41. DFT calculated structural models. a Relaxation structure of Ru/TiO_2 used to calculate OER reaction steps and **b-d** chemisorption models of *OH, *O, and *OOH on Ru site in Ru/TiO_2, respectively.

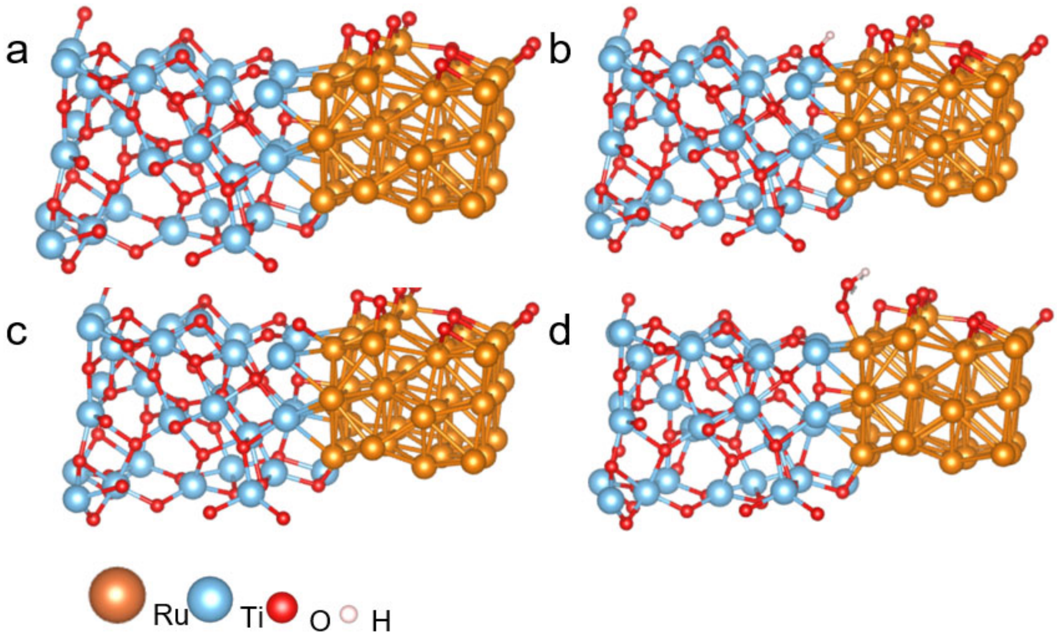

Supplementary Fig. 42. DFT calculated structural models. a Relaxation structure of Ru/Ti_4O_7 used to calculate OER reaction steps and **b-d** chemisorption models of *OH, *O, and *OOH on Ru site in Ru/Ti_4O_7, respectively.

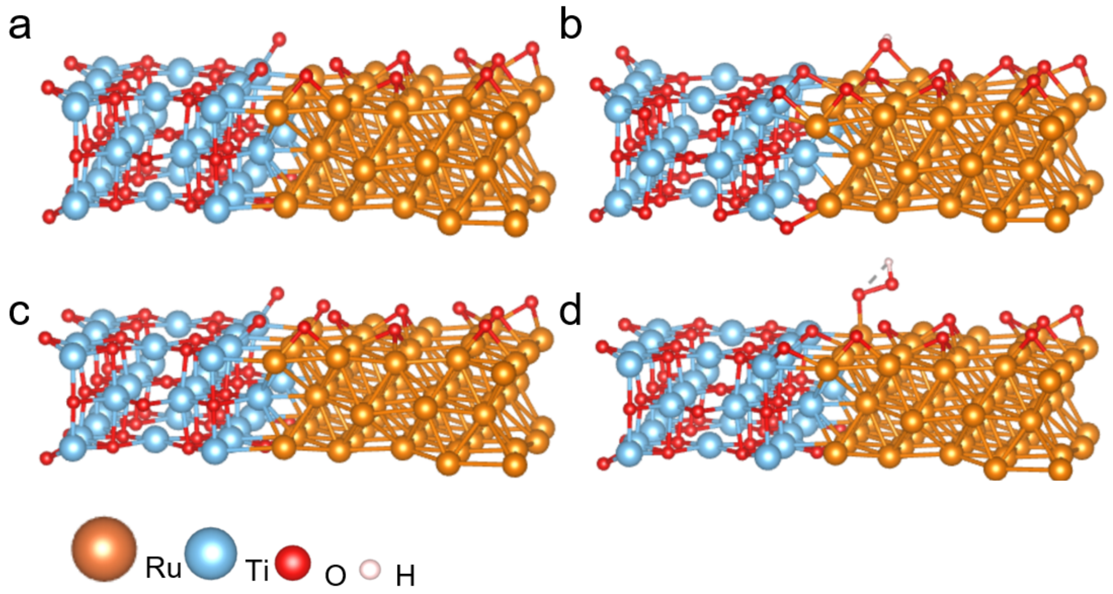

Supplementary Fig. 43. DFT calculated structural models. a Relaxation structure of Ru/TiO used to calculate OER reaction steps and **b-d** chemisorption models of *OH, *O, and *OOH on Ru site in Ru/TiO, respectively.

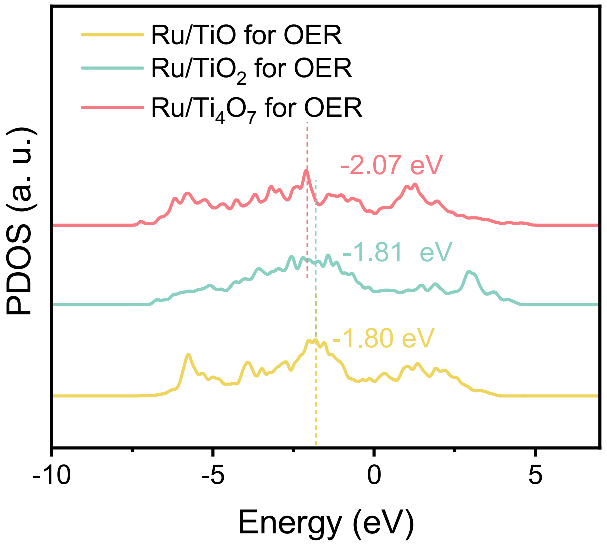

Supplementary Fig. 44. Calculation of the *d*-band structure based on the structural models for OER. Projected density of states (PDOS) of Ru 3*d* in the relaxation structures of Ru, Ru/Ti_4O_7, Ru/TiO_2, and Ru/TiO to calculate OER reaction steps.

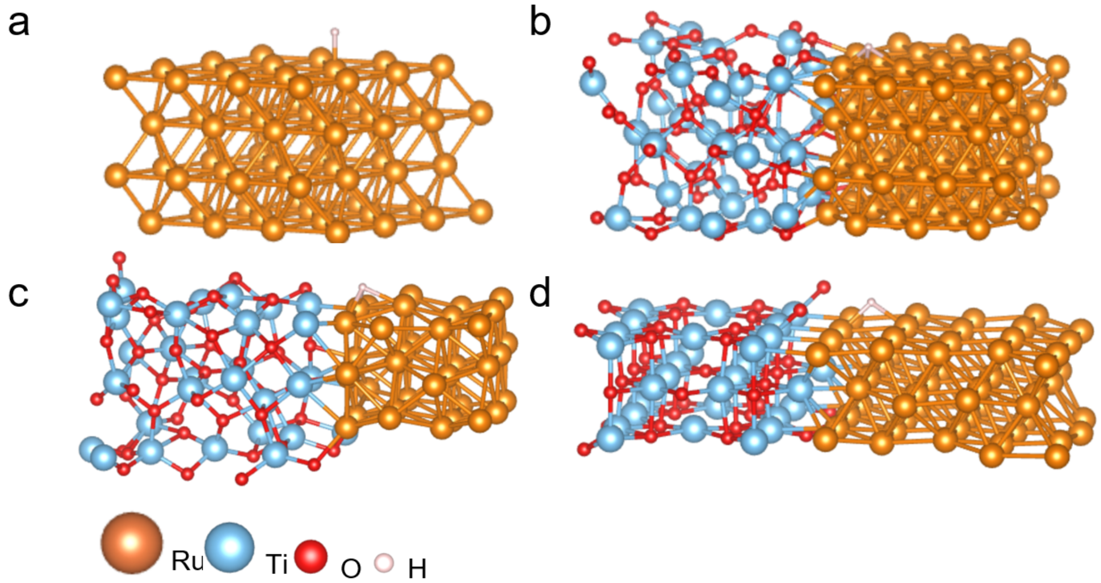

Supplementary Fig. 45. DFT calculated structural models. Chemisorption models of *H on Ru site in **a** Ru, **b** Ru/TiO_2, **c** Ru/Ti_4O_7, and **d** Ru/TiO, respectively.

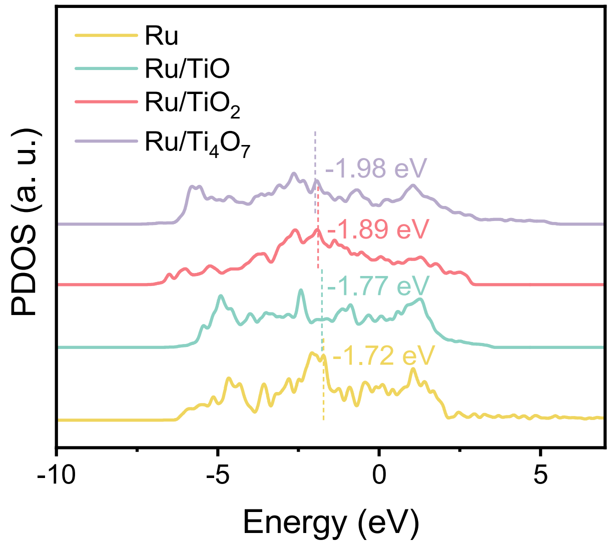

Supplementary Fig. 46. Calculation of the *d*-band structure based on the structural models for HER. PDOS of Ru 3*d* in the relaxation structures of Ru, Ru/Ti_4O_7, Ru/TiO_2, and Ru/TiO used to calculate HER reaction steps.

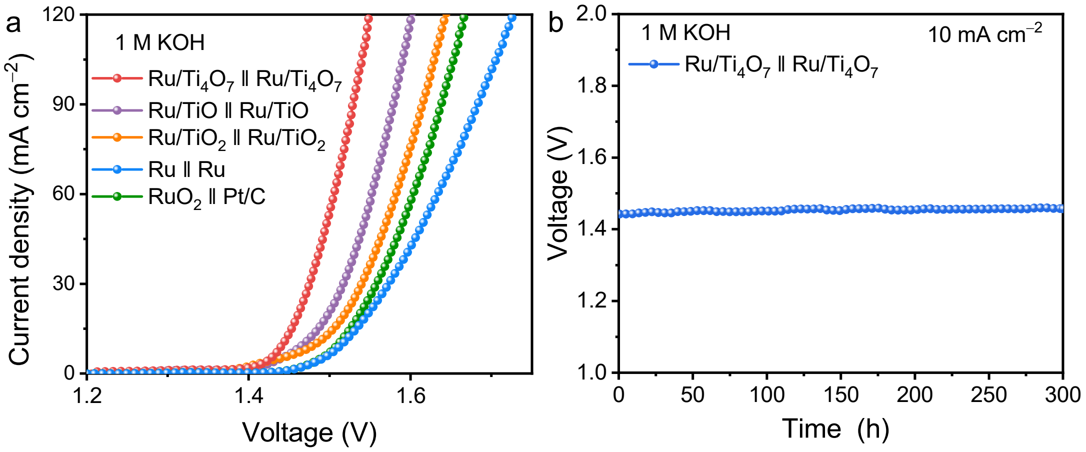

Supplementary Fig. 47. Activity and stability characterization of water splitting in the alkaline environment. a Polarization curves of Ru/TiO_2 ‖ Ru/TiO_2, Ru/Ti_4O_7 ‖ Ru/Ti_4O_7, Ru/TiO ‖ Ru/TiO, and RuO_2 ‖ Pt/C for water splitting in 1 M KOH, respectively. **b** Chronopotentiometry tests of Ru/Ti_4O_7 ‖ Ru/Ti_4O_7 at 10 mA cm^{-2} in 1 M KOH.

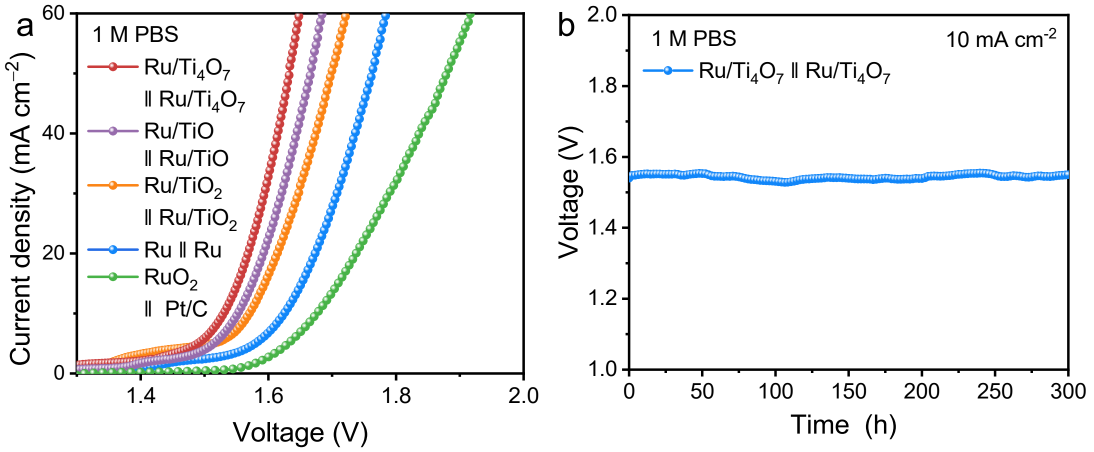

Supplementary Fig. 48. Activity and stability characterization of water splitting in the neutral environment. a Polarization curves of Ru/TiO_2 ‖ Ru/TiO_2, Ru/Ti_4O_7 ‖ Ru/Ti_4O_7, Ru/TiO ‖ Ru/TiO, and RuO_2 ‖ Pt/C for water splitting in 1 M PBS, respectively. **b** Chronopotentiometry tests of Ru/Ti_4O_7 ‖ Ru/Ti_4O_7 at 10 mA cm^{-2} in 1 M PBS.

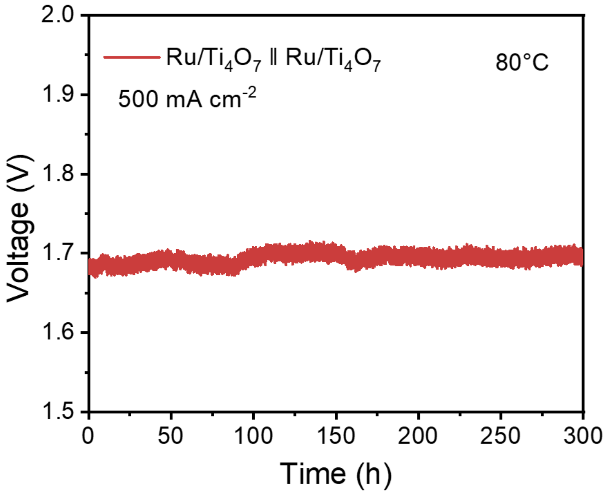

Supplementary Fig. 49. Stability tests of the PEM water electrolyzer under high-current-densities. Stability tests of Ru/Ti_4O_7 ‖ Ru/Ti_4O_7 for PEM electrolyzer at 500 mA cm^{-2}.

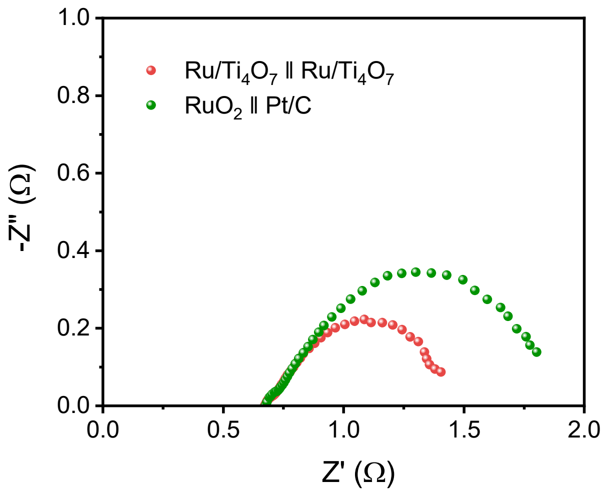

Supplementary Fig. 50. EIS tests of the PEM electrolyzer. EIS Nyquist curves of Ru/Ti_4O_7 ‖ Ru/Ti_4O_7, and commercial RuO_2 ‖ Pt/C for PEM water electrolysis.

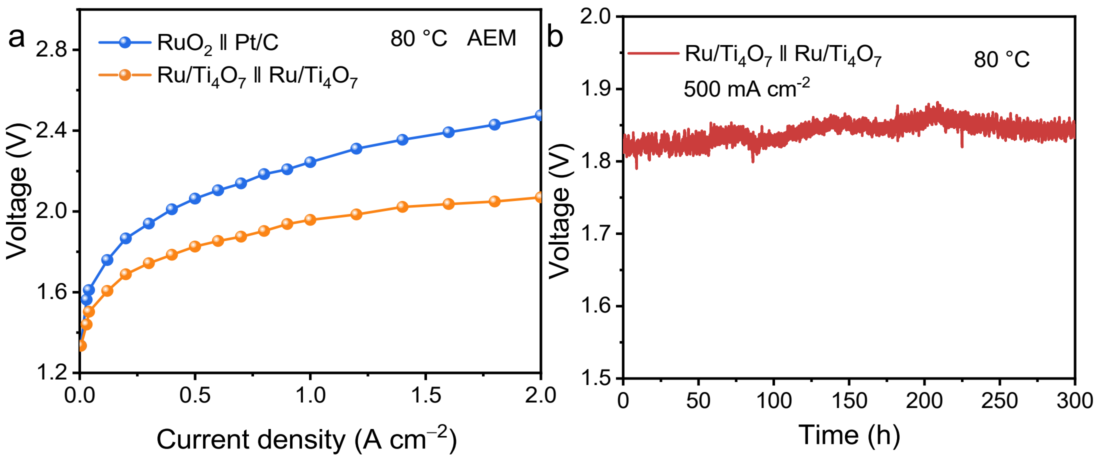

Supplementary Fig. 51. Activity and stability characterization of AEM electrolyzer. a Steady polarization curves of Ru/Ti_4O_7 ‖ Ru/Ti_4O_7 and RuO_2 ‖ Pt/C for anion exchange membrane (AEM) water electrolyzer using 1 M KOH as feedstock. **b** Chronopotentiometry tests of Ru/Ti_4O_7 ‖ Ru/Ti_4O_7 for AEM water electrolyzer at 500 mA cm^{-2}.

2 Supplementary Tables

Supplementary Table 1. The element content in Ru/TiO_2, Ru/Ti_4O_7, and Ru/TiO obtained using ICP-MS.

catalysts	Ti (wt.%)	Ru (wt.%)
Ru/TiO_2	53.13	21.31
Ru/Ti_4O_7	42.69	20.98
Ru/TiO	46.64	21.89

Supplementary Table 2. Comparison of OER performance of this work with that of pH-universal catalysts recently reported.

Catalysts	Noble metal content	Electrocatalytic activity						Reference
		0.5 M H_2SO_4		1 M KOH		1 M PBS		
		η_{10} (mV)	Stability@10 mA cm^{-2} (h)	η_{10} (mV)	Stability@10 mA cm^{-2} (h)	η_{10} (mV)	Stability@10 mA cm^{-2} (h)	
Ru/Ti_4O_7	20.98 wt% Ru	**150**	**500**	**180**	**300**	**220**	**300**	**This work**
$Ni\text{-}RuO_2$	15.52 wt% Ru	214	200	/	/	/	/	Nat. Mater. 2023, 22, 100.
$12Ru/MnO_2$	11.6 wt% Ru	161	200	/	/	/	/	Nat. Catal. 2021, 4, 1012.
$Ru\text{-}Pt_3Cu$	0.0163 mg_{Pt+Ru} cm^{-2}	280	28	/	/		/	Nat. Catal. 2019, 2, 304.
0.2Mo-PIO	21.73 wt% Ir	295	200	/	/	/	/	Nat. Commun. 2023, 14, 4127.
AuSA-MnFeCoNiCu LDH	1.1 wt% Au	/	/	213	700 h@100 mA cm^{-2}	/	/	Nat. Commun. 2023, 14, 6019.
Ir-NSG	7.33 wt% Ir	265	1.25	256	1.7	297	4.2	Nat. Commun. 2020, 11, 4246.
$RuIrZnO_x$	20 wt% Ru+Ir	240	24	220	24	/	/	Nat. Commun. 2019, 10, 4875.
Ru/Co–N–C	0.36 wt% Ru	232	20	276	20	400	20	Adv. Mater. 2022, 34, 2110103.
$Ru@V\text{-}RuO_2/C$ HMS	0.28 mg cm^{-2}	176	15	201	15	/	/	Adv. Mater. 2023, 35, 2206351.
FRNO/CC	0.527 mg cm^{-2}	200	100	260	100	/	/	Adv. Energy Mater. 2023, 13, 2300174.

RuCu NSs/C	/	236	15	234	20	/	/	Angew. Chem. Int. Ed. 2019, 58, 13983–13988.
Nicluster-Ru NWs	20 wt% Ru	205	10	194	10	/	/	Energy Environ. Sci., 2021, 14, 3194–3202.
BPIr_be	0.13 mg cm^{-2}	290	500	290	500	620	/	Adv. Mater. 2021, 33, 2104638.
IrO_2/V_2O_5	19.70 wt% Ir	266	20	283	20	329	20	Adv. Sci. 2022, 9, 2104636.
Ir-COP	9.81 wt% Ir	242	36	230	36	/	/	Adv. Funct. Mater. 2023, 33, 2211192.
Ir@Ni-NDC	9.72 wt% Ir	219	/	210	/	296	/	Angew. Chem. Int. Ed. 2023, 62, e202302220.
$Pd/NiFeO_x$	/	169	50	180	50	310	50	Adv. Funct. Mater. 2021, 31, 2107181.
a/c-RuO_2	/	220	20 h@50 mA cm^{-2}	235	/	287	/	Angew. Chem. Int. Ed. 2021, 60, 18821–18829.

Supplementary Table 3. Comparison of HER performance of this work with that of pH-universal catalysts recently reported.

Catalysts	Noble metal content	Electrocatalytic activity						Reference
		0.5 M H_2SO_4		1 M KOH		1 M PBS		
		η_{10} (mV)	Stability@10 mA cm^{-2} (h)	η_{10} (mV)	Stability@10 mA cm^{-2} (h)	η_{10} (mV)	Stability@10 mA cm^{-2} (h)	
Ru/Ti_4O_7	20.98 wt% Ru	**8**	**500**	**14**	**300**	**27**	**300**	**This work**
s-Pt/1T′-MoS_2	12.20 wt% Pt	19	240 h@1500 mA cm^{-2}	/	/	/	/	Nature 2023, 621, 300.
Pt_{1}/OLC	0.27 wt% Pt	38	100 h@40 mA cm^{-2}	/	/	/	/	Nat. Energy 2019, 4, 512.
Li-$Pd_3P_2S_8$	51.10 wt% Pd	91	13 h@20 mA cm^{-2}	/	/	/	/	Nat. Catal. 2018, 1, 460.
B-Os Aerogel	99.3 wt% Os	12	202	19	20	33	20	Nat. Commun. 2022, 13, 1143.
Ru@C_2N	0.285 mg cm^{-2}	22	/	17	/	/	/	Nat. Nanotech. 2017, 12, 441.
Ir-NSG	7.33 wt% Ir	17	11	18.5	17.7	16.8	1.7	Nat. Commun. 2020, 11, 4246.
$RuIrZnO_x$	20 wt% Ru+Ir	13	24	14	24	/	/	Nat. Commun. 2019, 10, 4875.
Pt_3Fe/NMCS-A	10.3 wt% Pt	13	10	29	10	48	10	Adv. Mater.2023, 35, 2303030.
Ru/Co–N–C	0.36 wt% Ru	17	20	19	20	87	20	Adv. Mater. 2022, 34, 2110103.

Ru@V-RuO_2/C HMS	0.28 mg cm^{-2}	47	15	6	15	/	/	Adv. Mater. 2023, 35, 2206351.
RuCu NSs/C	/	20	/	19	/	/	/	Angew. Chem. Int. Ed. 2019, *58*, 13983.
FRNO/CC	0.527 mg cm^{-2}	30	80	82	80	/	/	Adv. Energy Mater. 2023, 13, 2300174.
Nicluster-Ru NWs	20 wt% Ru	20	10	17	10	/	/	Energy Environ. Sci., 2021, 14, 3194–3202.
BPIr_be	0.13 mg cm^{-2}	26	100	1.98	100	329	/	Adv. Mater. 2021, 33, 2104638.
Ir-COP	9.81 wt% Ir	12.3	100	14.5	100	/	/	Adv. Funct. Mater. 2023, 33, 2211192.
Ir@Ni-NDC	9.72 wt% Ir	41	/	19	/	31	/	Angew. Chem. Int. Ed. 2023, 62, e202302220.
RuO_2-300Ar	0.2 mg cm^{-2}	16	/	17	300	29	/	Energy Environ. Sci. 2021, 14, 5433.
Ru@1T-MoS_2-MXene	15 wt% Ru	44	160	42	100	106	100	Adv. Funct. Mater. 2023, 33, 2212514.
Ru@Ni-MOF	2.3 wt% Ru	37	24 h @ 100 mA cm^{-2}	22	24 h@ 100 mA cm^{-2}	52	24 h @ 100 mA cm^{-2}	Angew. Chem. Int. Ed. 2021, 60, 22276–2282.

Supplementary Table 4. The parameters of the EIS data fitting of Ru/TiO_2, Ru/Ti_4O_7, and Ru/TiO for HER.

Catalysts	η (mV)	R_s (Ω)	R_1 (Ω)	R_2 (Ω)
Ru/TiO_2	−10	1.85	0.85	2.2
	−20	1.85	0.84	1.43
	−30	1.92	0.83	1.03
	−40	1.89	0.81	0.82
	−50	1.85	0.76	0.7
Ru/Ti_4O_7	−10	1.68	0.65	2.15
	−20	1.68	0.61	1.28
	−30	1.68	0.62	0.95
	−40	1.68	0.61	0.77
	−50	1.68	0.58	0.65
Ru/TiO	−10	1.92	0.62	2.60
	−20	1.92	0.59	1.59
	−30	1.93	0.58	1.19
	−40	1.84	0.56	0.95
	−50	1.84	0.55	0.83

Supplementary Table 5. Adsorption free energy of OER intermediates at U=0.

Catalysts	ΔG_{*OH} (eV)	ΔG_{*O} (eV)	ΔG_{*OOH} (eV)
Ru/Ti_4O_7	0.703	1.950	3.380
Ru/TiO_2	0.435	1.977	3.315
Ru/TiO	−0.482	0.808	2.624

Supplementary Table 6. Adsorption free energy changes of OER intermediates at U=0.

Catalysts	ΔG_1 (eV)	ΔG_2 (eV)	ΔG_3 (eV)	ΔG_4 (eV)	ΔG (eV)
Ru/Ti_4O_7	0.703	1.247	1.431	1.540	1.540
Ru/TiO_2	0.435	1.541	1.338	1.605	1.605
Ru/TiO	−0.482	1.290	1.816	2.296	2.296

Supplementary Table 7. Adsorption free energy of HER intermediates at U=0.

Catalysts	ΔG_{*H} (eV)
Ru	−0.745
Ru/Ti_4O_7	0.123
Ru/TiO_2	−0.171
Ru/TiO	−0.446

Supplementary Table 8. Comparison of the acidic water splitting performance of this work with that of the noble metal based catalysts recently reported.

Catalysts	Electrocatalytic activity						Reference
	0.5 M H_2SO_4		1 M KOH		1 M PBS		
	Voltage @ 10 mA cm^{-2} (V)	Stability @ 10 mA cm^{-2} (h)	Voltage @ 10 mA cm^{-2} (V)	Stability @ 10 mA cm^{-2} (h)	Voltage @ 10 mA cm^{-2} (V)	Stability @ 10 mA cm^{-2} (h)	
Ru/Ti_4O_7	1.44	300	1.44	300	1.53	300	**This work**
Ir-NSG	1.42	24	1.45	24	1.53	24	Nat. Commun. 2020, 11, 4246.
$RuIrO_x$	1.45	24	1.47	24	1.49	24	Nat. Commun. 2019, 10, 4875.
CPF-Fe/Ni	1.44	120	1.57	120	/	/	Nat. Commun. 2023, 14, 1792.
RuCu NSs/C	1.49	15	1.49	40	/	/	Angew. Chem. Int. Ed. 2019, 58, 13983–13988.
Ru/Co–N–C	1.49	/	1.5	/	/	/	Adv. Mater. 2022, 34, 2110103.
Ru@V-RuO_2/C HMS	1.47	25	1.44	25	1.47	25	Adv. Mater. 2023, 35, 2206351.
Nicluster-Ru NWs	1.45	/	1.44	/	/	/	Energy Environ. Sci. 2021, 14, 3194–3202.
BPIr_be	1.57	5	1.54	15	2.11	/	Adv. Mater. 2021, 33, 2104638.
IrO_2/V_2O_5	1.5	30	1.49	30	1.65	30	Adv. Sci. 2022, 9, 2104636.
Ir@Ni-NDC	1.54	35	1.46	100	1.59	30	Angew. Chem. Int. Ed. 2023, 62, e202302220.
HP-Ru/C	1.58	20	1.61	20	1.61	20	Appl. Catal. B-Environ. 2021, 294, 120230.

Supplementary Table 9. Comparison of the PEM performance of this work with that of the Ru based catalysts recently reported.

Catalysts	E (V)@J (A cm^{-2})	Stability (h) @J (A cm^{-2})	Reference
Ru/Ti_4O_7	**1.69@0.5**	**300@0.5**	**This work**
Ni-RuO_2	1.78@0.5	1000@0.2	Na®t. Mater. 2023, 22, 100.
SS Pt-RuO_2 HNSs	\	100@0.1	Sci. Adv. 2022, 8, eabl9271.
$Nb_{0.1}Ru_{0.9}O_2$	1.69@1	100@0.3	Joule 7, 2023, 558.
$RuCoO_x$	1.56@0.2	10@0.1	J. Am. Chem. Soc. 2023, 145, 17995.
Y_2MnRuO_7	1.75@1	24@0.2	Nat. Commun. 2023, 14, 2010.
$W_{0.2}Er_{0.1}Ru_{0.7}O_{2-\delta}$	\	120@0.1	Nat. Commun. 2020, 11, 5368.
Ru/Co–N–C-800 C	\	330@0.45	Adv. Mater. 2022, 34, 2110103.
$Nd_{0.1}RuO_x$/CC	1.595@0.05	50@0.01	Adv. Funct. Mater. 2023, 33, 2213304.
$Ru_{0.6}Cr_{0.4}O_2$	\	12@0.1	Small Methods 2022, 6, 2200636
